# Genomic and *Cis*‐Regulatory Basis of a Plastic C_3_‐C_4_ Photosynthesis in *Eleocharis Baldwinii*

**DOI:** 10.1002/advs.202415681

**Published:** 2025-05-30

**Authors:** Lu Chen, Yanfeng Jia, Zaihui Zhou, Qiying Jiang, Jianlin Liu, Jianhui Gu, Chuan Chen, Shujing Cheng, Jinfang Chu, Xin Liu, Yongjun Lin, Xiang Li

**Affiliations:** ^1^ Laboratory of Advanced Breeding Technologies Institute of Genetics and Developmental Biology (IGDB) Chinese Academy of Sciences Beijing 100101 China; ^2^ IGDB‐BGI Joint Center for Multiomics Beijing 100101 China; ^3^ National Key Laboratory of Crop Genetic Improvement and National Center of Plant Gene Research College of Life Science and Technology Huazhong Agricultural University Wuhan 430070 China; ^4^ University of Chinese Academy of Sciences Beijing 100049 China; ^5^ BGI Research Beijing 102601 China; ^6^ National Centre for Plant Gene Research (Beijing) State Key Laboratory of Seed Innovation Institute of Genetics and Developmental Biology (IGDB) Chinese Academy of Sciences Beijing 100101 China

**Keywords:** C_4_ photosynthesis, photosynthetic plasticity, regulatory elements, single‐cell multi‐omics

## Abstract

C_4_ photosynthesis is a crucial trait for efficient carbon fixation; however, the genomic basis and environmental influences on its formation remain under explored. Here, we examined *Eleocharis baldwinii*, a sedge that exhibits photosynthetic plasticity with C_3_‐like traits underwater and C_4_‐like traits on land, to expand knowledge of C_4_ photosynthesis. A gap‐free allopolyploid genome was assembled and a cellular expression atlas is reconstructed by applying single‐nucleus RNA‐seq.Subgenome dominance influencing expression of C_4_ enzyme genes was detected across cell types. *cis*‐regulatory patterns linked to the C_4_ pathway, Kranz anatomy specialization, and environmental responses are evaluated using single‐nucleus transposase‐accessible chromatin sequencing and found that the formation of the C_4_ pathway involves a few environmentally triggered, cell‐specialized *cis*‐regulatory elements. Comparative analyses between *E. baldwinii* and maize further implied that *cis*‐regulator families for Kranz anatomy specialization are conserved across species, whereas those for C_4_ metabolism differ, underscoring the genetic diversity underlying convergent traits. This study provides genomic and regulatory insights into how plants evolve C_4_ photosynthesis, especially regarding the effects of environmental factors.

## Graphical abstract

The sedge *Eleocharis baldwinii* remarkably switches between C_3_‐like and C_4_‐like photosynthesis in response to environmental conditions. Combining gap‐free genome assembly with single‐cell multi‐omics, this study uncovers how subgenome dominance and cell‐specific *cis*‐regulation enable C_4_ photosynthesis. Comparative analyses reveal distinct genomic and regulatory basis of C4 photosynthesis between sedges and grasses, offering new perspectives on photosynthetic evolution.

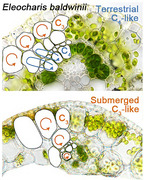

## 1 Introduction

C_4_ photosynthesis functions as a complex trait with a high carbon fixation rate. This relies on a CO_2_‐concentrating mechanism within the specialized leaf anatomy known as Kranz, which was discovered ≈60 years ago.^[^ ^1^, ^2^ ^]^ Global surveys and phylogenetic analyses demonstrated that this sophisticated trait has evolved independently in 22 angiosperm families with over 60 known origins.^[^ ^3^, ^4^, ^5^, ^6^ ^]^ Such widespread convergence highlights the critical adaptive role of C_4_ photosynthesis in response to historical environmental alterations. Given its potential to improve C_3_ crop production and its criticality in understanding convergent evolution, anatomical structure comparisons between C_3_ and C_4_ plants and molecular investigations of C_4_ enzymes have been of great interest in the last decades.^[^ ^7^, ^8^, ^9^, ^10^ ^]^ However, a comprehensive understanding of the underlying molecular mechanisms is lacking.

In typical C_4_ plants, the Kranz anatomy provides the structural foundation for the CO_2_‐concentrating mechanism, consisting of mesophyll cells (MC) and enlarged bundle sheath cells (BSC) surrounding the vascular bundles. In MC, phosphoenolpyruvate carboxylase (PEPC) catalyzes the initial CO_2_ fixation into four‐carbon compounds.^[^ ^11^, ^12^ ^]^ These compounds subsequently diffuse into the BSC, where they are decarboxylated, releasing CO_2_ facilitated by either NADP‐malic enzyme (NADP‐ME), NAD‐malic enzyme (NAD‐ME), or phosphoenolpyruvate carboxykinase (PPCK), forming three C_4_ photosynthesis subtypes. The resulting increased CO_2_ concentration around ribulose‐1,5‐bisphosphate carboxylase (RuBisCO) minimizes photorespiration and increases the efficiency of the Calvin–Benson cycle, thereby boosting carbon fixation.^[^ ^13^, ^14^, ^15^ ^]^ Despite the conserved functions of C_4_ photosynthesis across plants, the factors driving its evolution are complicated. The rise of C_4_ grasses has been associated with decreasing atmospheric CO_2_ levels since the Oligocene alongside drying climates.^[^ ^22^, ^23^ ^]^ This adaptation can be attributed to the increased CO_2_ concentration around RuBisCO in C_4_ plants. Furthermore, C_4_ plants are predominantly found in hot habitats^[^ ^4^, ^5^, ^24^ ^]^ where high temperatures promote stomatal closure to reduce water transpiration, consequently lowering mesophyll‐interstitial CO_2_ levels. Therefore, C_4_ photosynthesis is well‐suited for handling such situations. Despite the identification of environmental factors, the genomic basis of their collective influence on the C_4_ plant evolution remains elusive.

The coding genes and regulatory elements involved in C_4_ photosynthesis vary despite the similar enzymatic machinery across plant lineages,^[^ ^9^ ^]^ emphasizing the complexity and plasticity of their fundamental metabolism. For example, the C_4_ enzyme gene *PEPCs* in grasses evolved independently at least eight times from the same non‐C_4_ *PEPC* ancestor^[^ ^16^ ^]^; *cis*‐regulatory motifs regulating *PEPCs* expression, such as mesophyll expression module 1 (*MEM1*) in *Flaveria* ^[^ ^19^, ^20^ ^]^ and conserved nucleotide sequences (*CNSs*) in panicoid grasses, evolved independently.^[^ ^21^ ^]^ Contrastingly, conserved transcription factor families that regulate the metabolism of photosynthesis and formation of Kranz anatomy, such as G2/GLK and SCR/SHR, respectively, have been repeatedly acquired in various plants.^[^ ^9^, ^17^, ^18^ ^]^ However, the genomic basis of the formation of C_4_ photosynthesis still remains largely unexplored.

Furthermore, the discovery of plants with C_3_‐C_4_ intermediates, such as proto‐Kranz, C_2_, and C_4_‐like forms,^[^ ^3^, ^5^ ^]^ provides a detailed view of gradual C_4_ photosynthesis evolution.^[^ ^9^, ^25^, ^26^, ^27^ ^]^ Several species of *Eleocharis* in the Cyperaceae family have been reported to exhibit photosynthetic plasticity between C_3_ or C_3_‐like forms under submerged conditions and C_4_ or C_4_‐like forms in terrestrial environments.^[^ ^28^, ^29^, ^30^ ^]^ Additionally, these photosynthetic forms can also be shifted by abscisic acid (ABA),^[^ ^31^, ^32^ ^]^ gibberellic acid (GA),^[^ ^33^ ^]^ or saline treatments.^[^ ^34^ ^]^ Herein, we focused on *Eleocharis baldwinii*, a species exhibiting photosynthetic plasticity between C_3_‐like and C_4_‐like forms, providing an excellent system to understand how environmental factors influence the gradual C_4_ photosynthesis formation. We assessed the evolutionary history of photosynthetic plasticity by assembling its gap‐free allotetraploid genome and comparing it with the recent genome assembly of *Eleocharis vivipara* (*E. vivipara*).^[^ ^30^ ^]^ By applying single‐nucleus transcriptome (snRNA‐seq) and transposase‐accessible chromatin sequencing (snATAC‐seq), we evaluated the differences in gene expression and cis‐regulatory patterns for C_4_ photosynthesis, Krans specialization, and environmental responses. Our results revealed cellular subgenome dominance in C_4_ photosynthesis, with distinct subgenomes driving high gene expression in various cell types. C_4_ photosynthesis indicates the involvement of several environmentally triggered *cis*‐regulatory elements, providing new evidence for understanding the molecular basis of C_3_‐C_4_ photosynthetic plasticity in *Eleocharis*. Comparative analyses indicated that grasses and *E. baldwinii* evolved *cis*‐regulators of distinct families for performing C_4_ metabolism but shared similar *cis*‐regulator families for Kranz anatomy specialization. These findings expand our understanding of the diverse molecular evolutionary mechanisms underlying C_4_ photosynthesis across different plant lineages.

## 2 Results

### 2.1 Environments Drive the Developmental Fates into C_3_ or C_4_ Photosynthesis

**Figure 1:**
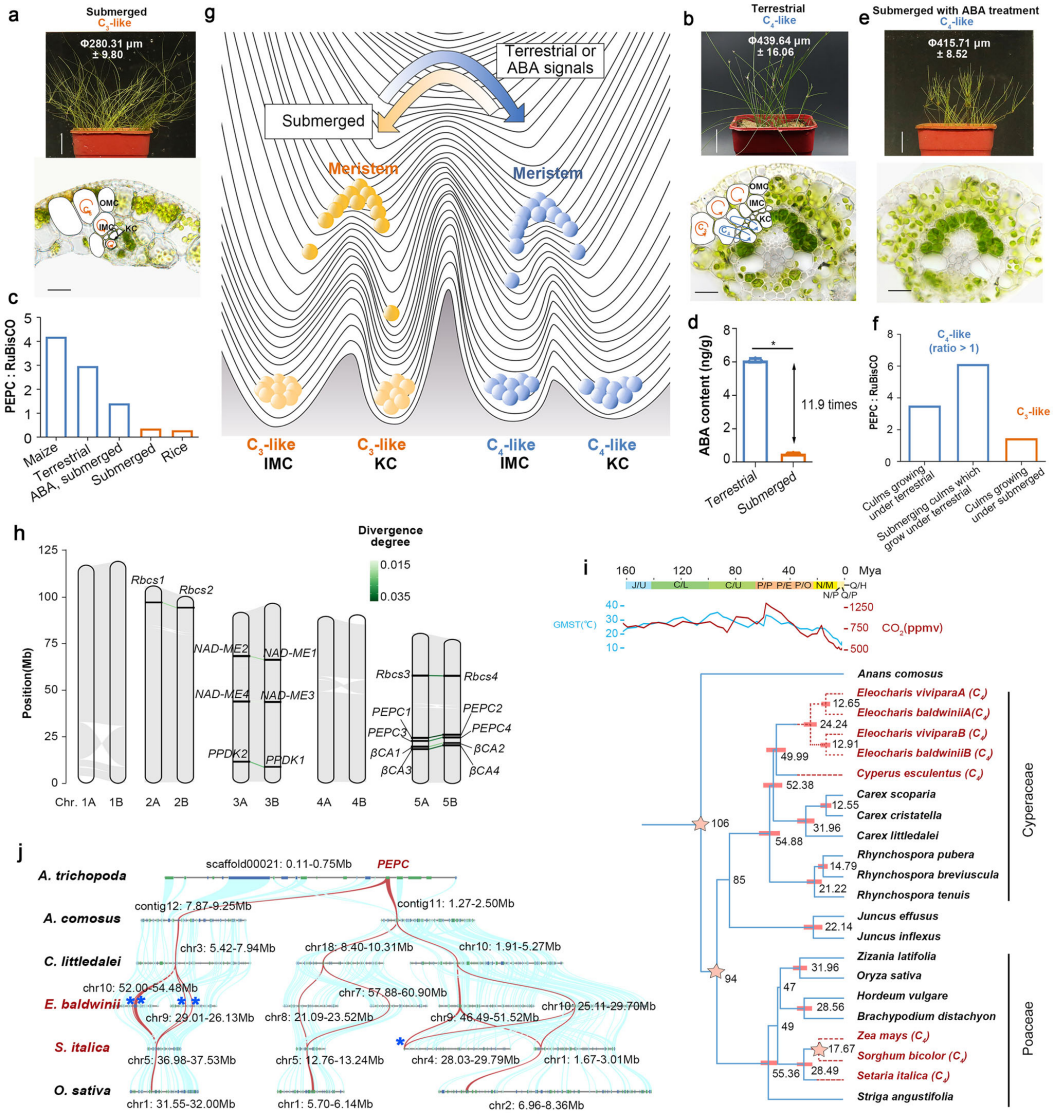
The photosynthetic plasticity in *Eleocharis baldwinii* and its evolutionary trajectory. a,b,e) The photographs of plants (upper) and culm vascular bundle anatomy (lower) growing under varying conditions, including submerged environments with fresh water (a), terrestrial (b), and submerged environments with 5 µm ABA (e). For the plant photograph, scale bar = 2 cm. The thickness of the plant culm is presented as means ± standard deviation (SD), n = 8. For the culm anatomy, scale bar = 20 µm. c) The PEPC / RuBisCO activity ratios of culms in *E. baldwinii*, leaves in maize and rice. n ≥ 3. d) The ABA content of the terrestrial and submerged culms of *E. baldwinii*. Data are presented as means ± SD in (d), n = 3, and statistical analyses were performed using Student's *t*‐test: *, *P* < 0.0001. f) The ratio of phosphoenolpyruvate carboxylase (PEPC)/ ribulose‐1,5‐bisphosphate carboxylase (RuBisCO) activities when submerging the mature culms which grow under terrestrial conditions; n = 3. g) A model for cellular development fates for C_3_‐ or C_4_‐like forms. Alternative environments may shift the potentials of meristems to developing C_3_‐ or C_4_‐like forms. h) Inter‐chromosomal synteny of the *E. baldwinii* genome, with an exhibition of the C_4_ core genes along the chromosomes. i) Phylogenetic relationships of the 20 representative monocots, including the distinguished sub‐genomes of *E. baldwinii* and *E. vivipara*, rooted with *Anans comosus*. The complete phylogenetic tree adding 14 eudicots and the ancient angiosperm *Amborella trichopoda* is displayed in Figure S5 (Supporting Information). The top axis reveals the geological timescale, with the initials of Period/Epoch. J/U represented Jurassic/Upper, C/L: Cretaceous/Lower, C/U: Cretaceous/Upper, P/P: Paleogene/Paleocene, P/E: Paleogene/Eocene, P/O: Paleogene/Oligocene, N/M: Neogene/Miocene, N/P: Neogene/Pliocene, Q/P: Quaternary/Pleistocene Q/H: Quaternary/Holocene. The line plot indicates the CO_2_ concentration and temperature along with time.^[^ ^52^ ^]^ In the phylogenetic tree, stars and bars indicate the calibration nodes and estimated confidence intervals, respectively. The red dashed lines indicate the possible stage for C_4_ photosynthesis emergence. j) Synteny analysis of the C_4_ *PEPCs* in the monocots and their ancestor gene originating from *Amborella trichopoda*. The red lines highlight the *PEPC* collinearity. The blue asterisks indicate the C_4_‐specific *PEPC* in the monocots.

We compared the physiological characteristics and anatomical structures under various environmental conditions to confirm photosynthetic plasticity in *E. baldwinii*.^[^ ^28^, ^31^, ^32^ ^]^ In terrestrial thick culms, we observed larger Kranz cells (KC) with abundant chloroplasts (**Figure** **1**a,b; Figure S1a, Supporting Information) and a higher PEPC/RuBisCO activity ratio (3.0) than in the submerged culms (0.4) (Figure 1c; Figure S1b, Supporting Information). Immunological analysis showed that RuBisCO and NAD‐ME were enriched in the KC of terrestrial culms (Figure S1c, Supporting Information), indicating the establishment of the NAD‐ME subtype C_4_ pathway. However, these enzymes were not exclusively present in KC but also weakly in the MC, coexisting with residual C_3_ activity, thereby supporting the previous findings that terrestrial culms exhibit a C_4_‐like form.^[^ ^28^, ^35^ ^]^ Since the C_4_ pathway is not established in the submerged culms, we classified them as C_3_‐like forms, which we considered to be a more accurate characterization than the previously proposed C_3_‐C_4_ forms.^[^ ^28^, ^35^ ^]^ Notably, we found an ultra‐high level (11.9 folds than that under submerged conditions) of ABA, a key endogenous phytohormone in response to dry conditions, in terrestrial culms using liquid chromatography‐tandem mass spectrometry (LC‐MS/MS) analysis (Figure 1d). After treating submerged culms with ABA solution for one month, we detected C_4_‐like traits, including a high KC area, enhanced PEPC/RuBisCO activity, and cell‐enriched distribution of metabolic enzymes (Figure 1e; Figure S1a–c, Supporting Information). These findings support the previous observations that terrestrial ABA signals promote the construction of C_4_ photosynthesis.^[^ ^31^, ^32^ ^]^

We investigated the culms within a few days of environmental changes to dissect the photosynthetic switch process (Figure S1d, Supporting Information). C_3_‐like thin culms withered after draining, and new C_4_‐like culms emerged from the plant base. Conversely, mature C_4_‐like culms that grew under terrestrial conditions remained vibrant after submergence, maintaining the C_4_ metabolic pathway with a PEPC/RuBisCO activity ratio > 1 (Figure 1f), whereas new C_3_‐like thin culms developed from the meristem. These observations suggest that mature culms maintain stable photosynthetic forms and that terrestrial or submerged conditions drive the potential of the meristem to form C_4_‐ or C_3_‐like photosynthesis (Figure 1g).

### 2.2 No‐Gap *E. baldwinii* Genome Assembly

Accurate genome assembly is critical for identifying genetic elements and evaluating their evolutionary history. Genome survey analysis showed that *E. baldwinii* is a tetraploid, consisting of ≈50% repeat sequences, with a haploid genome size of 490 Mb (n = 2x = 980 Mb), which was slightly larger than the estimated 936 Mb from flow cytometry (Figure S2a–c, Supporting Information). Additionally, we found a high proportion of *aabb* (6.64%) k‐mers and a low proportion of *aaab* (0.0331%) k‐mers, indicating *E. baldwinii* is an allotetraploid (Figure S2b and Table S1, Supporting Information). We performed *de novo* assembly of 1085 consensus contigs with an N50 of 86.4 Mb, using 33 Gb (N50 = 20 Kb) of PacBio highly accurate long high‐fidelity (HiFi) long reads. Subsequently, 206 Gb of the Hi‐C data were generated to anchor these contigs to chromosomes (Figure S2d, Supporting Information). After gap closure, we obtained ten complete chromosomes of 971Mb (Figure 1h; and Table S2, Supporting Information). Moreover, Hi‐C and collinearity analyses further supported that *E. baldwinii* is an allotetraploid (2n = 4x = 20; x = 5), with the same basic chromosome number as Cyperaceae^[^ ^36^, ^37^ ^]^ (Figure S2d,e, Supporting Information). The chromosomes from each subgenome were further distinguished using SubPhaser^[^ ^38^ ^]^ (Figures S2e and S3, Supporting Information). We evaluated the accuracy and completeness of the genome assembly by aligning 1.9 billion short reads to the genome, thereby achieving a high mapping rate (99.7%) with only 0.04% SNPs and 0.008% insertions and deletions (InDels) (Table S3, Supporting Information). The long‐terminal repeat assembly index (LAI) score of the assembled genome was 18.56. Next, we annotated 43113 protein‐coding gene models, with an average gene length of 4,016 bp and a coding sequence length of 1179 bp, demonstrating a higher consistency with other plants (Figure S4, Supporting Information). Moreover, 95.3% of the conserved single‐copy genes were found in 1614 benchmarking universal single‐copy orthologs of Embryophyta (BUSCOs; Figure S2f and Table S2, Supporting Information). Taken together, these findings indicate the high completeness and accuracy of the genome assembly and gene annotation. We found that the *E. baldwinii* genome comprised 60.05% repeat sequences (Table S4, Supporting Information), consisting of abundant long‐terminal‐repeat retrotransposons (LTR‐RTs, 7.83% copia. and 13.57% gypsy) and DNA transposons (helitrons, 15.35%) (Figure S2f, Supporting Information).

### 2.3 Evolutionary History of the Environment‐Inducible C_4_ Photosynthesis

We constructed phylogenetic trees for *E. baldwinii* and representative angiosperm species with C_3_ and C_4_ photosynthesis based on 1601 orthologous genes (phylogenetic trees including 20 monocot species in Figure 1i; full trees including 35 angiosperm species, see Figure S5, Supporting Information) to trace the evolutionary trajectory of environment‐inducible C_4_ photosynthesis. Our findings confirm a previously reported relationship in Cyperaceae, in which *Eleocharis* is a close relative of *Cyperus*.^[^ ^30^, ^39^ ^]^ We estimated an earlier divergence between *Eleocharis* and *Cyperus* at ≈50 million years ago (Mya) compared to the previously recognized 30 to 40 Mya.^[^ ^30^, ^39^ ^]^ This is possibly due to the comparison of *E. baldwinii* and *E. vivipara* subgenomes, clarifying the interspecies genetic variance. We observed a close relationship between the subgenomes of *E. baldwinii* and *E. vivipara* (A vs A and B vs B). Their homeologous subgenomes, A and B, diverged at ≈24 Mya (A vs B), and the same subgenomes between species (A vs A; B vs B) diverged at ≈13 Mya (Figure 1i). The Ks distributions of the two species further confirmed their similar polyploidization times (Figure S6, Supporting Information). Building upon the phylogenetic framework of C_4_ *Eleocharis* species,^[^ ^40^ ^]^ our genomic analyses provide new evidence for a close evolutionary relationship between these two C_3_‐C_4_ intermediate species.

We investigated *PEPCs* across species to examine the evolution of the C_4_ gene. We observed that all C_4_ plants had at least one *PEPC* with a serine residue corresponding to Ser780 in maize.^[^ ^11^, ^12^ ^]^ Contrastingly, other *PEPCs* in the C_4_ and C_3_ plants had alanine residues at that site. We then conducted a synteny analysis of the genomic blocks anchoring C_4_ *PEPCs* in *Setaria italica* (Poaceae) and *E. baldwinii* (Cyperaceae) (indicated by blue asterisks in Figure 1j). This was compared to their C_3_‐related species *Oryza sativa* and *Carex littledalei*, as the Poales plant *Ananas comosus*,^[^ ^41^ ^]^ and the ancestral angiosperm *Amborella trichopoda*.^[^ ^42^ ^]^ Our results suggest that these blocks originated from the same region of *A. trichopoda*, which then duplicated and diverged in Poales. Additionally, noncolinear *PEPCs* between *S. italic* and *E. baldwinii* were neo‐functionalized to establish the C_4_ pathways. This study provides comparative gene block evidence to support the previous conclusion detected by gene phylogeny analysis that various *PEPC* gene clades across plants evolved independently to establish the C_4_ pathways.^[^ ^16^ ^]^

### 2.4 Single‐Nucleus Transcriptome Atlas for the C_3_ and C_4_ Culms

**Figure 2:**
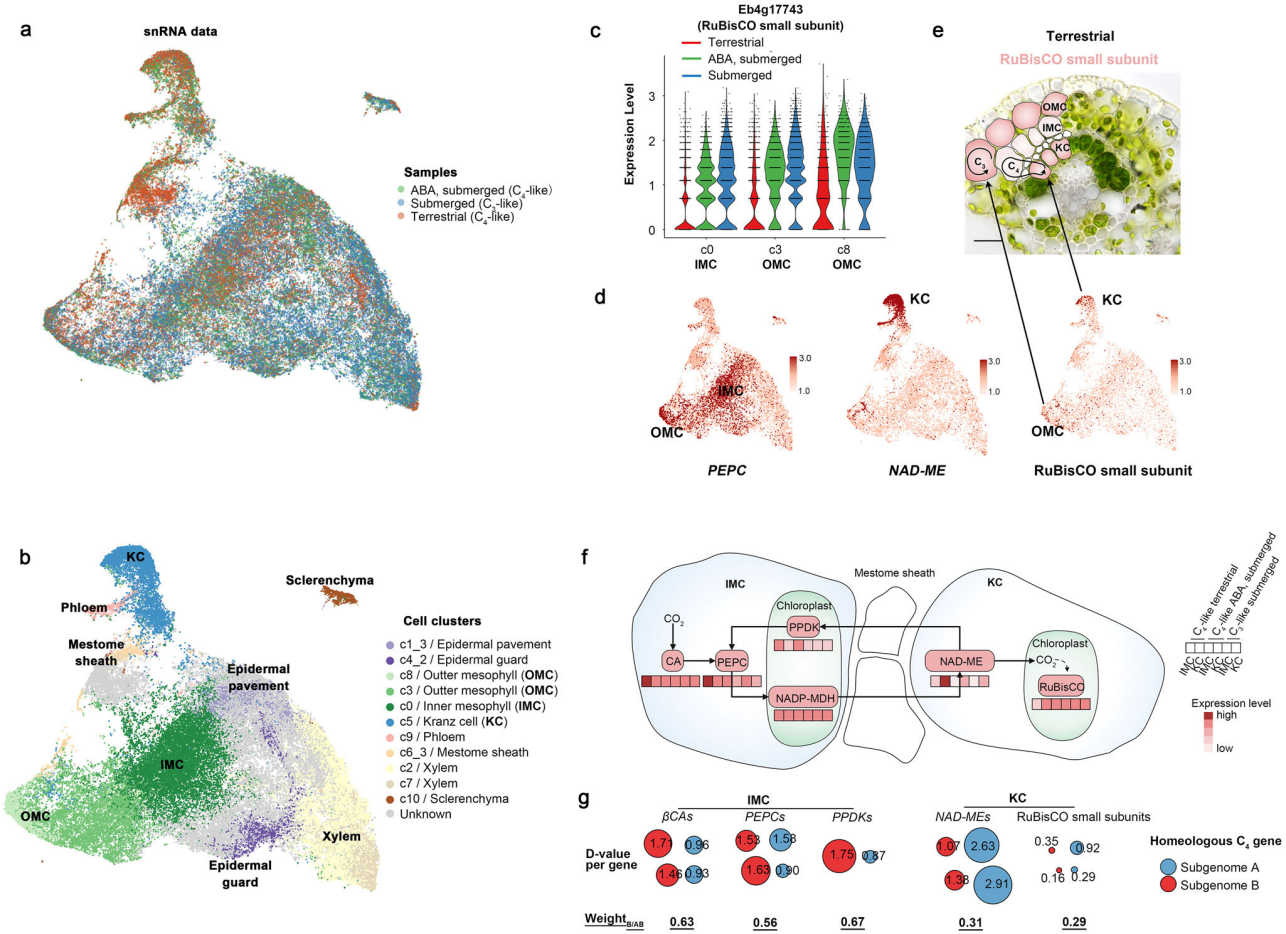
Single nucleus RNA‐seq reveals cellular expression of the C_4_ genes. a,b) The Uniform Manifold Approximation and Projection (UMAP) plots of single nucleus transcriptomes marked by the environment conditions of sample growth (a) and cell clusters (b). c) Expression level of RuBisCO small subunit in inner and outer mesophyll cells (IMC and OMC). d) The UMAP plots visualized single‐cell expressions of *PEPC*, *NAD‐ME*, and RuBisCO small subunit. e) A model indicating that OMC expressing RuBisCO independently fixes CO_2_ while IMC lacking RuBisCO may participate in the C_4_ pathway alongside KC. The red scale in the cells represents RuBisCO level enrichment. f) A schematic outlining the cell‐type enriched expression of the main enzyme genes for NAD‐ME C_4_ photosynthesis in *E. baldwinii*. The expression levels (red scale) are averaged across all paralogs for each enzyme. g) The D‐values between IMC and Kranz cells (KC) of each C_4_ gene under terrestrial conditions, colored by the subgenome where these genes are located. *D* − *value*  =  |*Exp_imc_* − *Exp_kc_* | Exp_imc_ represents the expression level of a C_4_ gene in the IMC under terrestrial conditions, whereas Exp_kc_ represents the expression level of the same gene in KC under terrestrial conditions. Expressional weights for homeologous genes in each subgenome were calculated. $Weight_{B/AB}=\frac{\sum D-value_{B}}{\sum D-value_{A}+\sum D-value_{B}}$ D‐value_A_ and D‐value_B_ represent the D‐values of homeologous genes in subgenomes A and B, respectively.

We performed single‐nucleus transcriptome sequencing (snRNA‐seq) on 12‐day‐after‐transplantation culms of *E. baldwinii* under three environments: ①terrestrial, and submerged environments ②with or ③without 5 µM ABA treatment. The snRNA‐seq data included 75202 cells (24830 from terrestrial culms, 17005 from submerged culms treated with ABA, and 33367 from submerged culms in freshwater), averaging 1232 unique molecular identifiers (UMIs) per cell (Figure S7a–d and Table S5, Supporting Information). High correlations in gene expression were noted among the biological replicates in the same environment (seven replicates for terrestrial culms, three for submerged ABA, and five for submerged freshwater). This indicated good repeatability of the snRNA‐seq datasets (**Figure** **2**a; Figure S7e–h, Supporting Information).

Following normalization and integration of cells across various conditions, the single‐nucleus data were categorized into 11 major clusters using an unsupervised model, with 1084–14368 cells per cluster. c1, c4, and c6 were further categorized into four, three, and four sub‐clusters (Figure S8a–c, Supporting Information), respectively. We annotated eight known cell types (Figure 2b; Figure S8d, Supporting Information) using 16 maker genes from known cell types in *Arabidopsis*, maize, and rice (See methods for details) (Table S6, Supporting Information): mesophyll cells (MC: c0, c3, c8), epidermal pavement (c1_3), mestome sheath (c6_3), xylem (c2, c7), Kranz cells (KC: c5), sclerenchyma (c10), phloem (c9), epidermal guard cells (c4_2), whereas cell types of other clusters or subclusters were unknown (c1_0,c1_1,c1_2, c4_0,c4_1, c6_0,c6_1, c6_2).

In typical C_4_ plants like maize, PEPC is present in the MC, and RuBisCO is present in the BSC. This created a CO_2_‐concentrating mechanism between the MC and BSC. *E. baldwinii* has two MC layers, including the outer (OMC) and inner mesophyll cells (IMC), and the C_4_ pathway was established only between the IMC and KC.^[^ ^35^ ^]^ The single‐nucleus transcriptome atlas clearly distinguished OMC (c3 and c8) and IMC (c0), with c3 and c8 demonstrating higher expression levels of the RuBisCO small subunit than c0 in terrestrial environments (Figure 2c,d). This indicated that OMC can independently fix CO_2_, whereas IMC may participate in the C_4_ pathway alongside KC (Figure 2e). In situ RNA hybridization of IMC/OMC‐enriched *PEPC* (*Eb9g39367*), OMC/KC‐enriched RuBisCO small subunit (*Eb9g40901*), and KC‐enriched NAD‐malic enzyme (*NAD‐ME*, *Eb6g28530*) confirmed the annotations of key cells for establishing the C_4_ pathway (Figure S9, Supporting Information). We then focused on IMC (c0) and KC (c5) to analyze their roles in the formation of the C_4_ pathway.

### 2.5 Cellular Expression and Subgenome Dominance of the C_4_ Genes

The high‐quality single‐nucleus data enabled the accurate evaluation of expression levels of all NAD‐ME C_4_ enzyme genes (Figure 2f; Figure S10 and Table S7, Supporting Information). Among IMC and KC across the three conditions, we observed terrestrial expressional enhancement of genes, such as β‐Carbonic anhydrases (*βCAs*), *PEPCs* in IMC and *NAD‐MEs* in KC, and terrestrial expressional inhibition of genes like RuBisCO small subunit in IMC. Furthermore, only pyruvate orthophosphate dikinases (*PPDKs*), but not the above genes, demonstrated high expression levels in IMC under terrestrial and ABA‐treated conditions. This suggests an additional mechanism for driving C_4_ photosynthesis beyond the ABA‐regulating pathway. Moreover, we noted that NADP‐malate dehydrogenases (*NADP‐MDHs*) showed non‐discriminatory expression levels in IMC and KC, as well as in other C_4_ enzyme genes such as aspartate transaminase (*AspAT*) and alanine transaminase (*AlaAT*) (Table S7, Supporting Information).

As *E. baldwinii* is an allotetraploid, we subsequently evaluated whether one subgenome dominated C_4_ gene expression. We analyzed the gene selective pressures, gene family compositions, and transposon distributions per chromosome of *E. baldwinii*. The results revealed a high similarity between homeologous chromosomes (Figure S11, Supporting Information). Additionally, we compared the selective pressures of the C_4_ genes between subgenomes (Table S8, Supporting Information). The RuBisCO small subunit demonstrated dominance in one subgenome. Combined with single‐cell transcriptome data, we compared the expression levels of homeologous genes encoding C_4_ enzymes between the subgenomes. We identified genes with high expression in specific cell types, including four *βCAs*, four *PEPCs* (with C_4_‐specific serine residue), and two *PPDKs* in IMC, as well as four *NAD‐MEs* and four RuBisCO small subunits in KC (highlighted in yellow in Table S7, Supporting Information)—where paired homeologous genes were identified for each gene. Subsequently, we calculated the difference in expression (D‐value) of each gene between the IMC and KC under terrestrial conditions. In the differently expressed C_4_ genes, those with higher expression in the IMC demonstrated larger D‐values for homeologous genes within subgenome B, such as weights_B/AB_ (within subgenome B/whole genome) of 0.63 for *βCAs*, 0.56 for *PEPCs*, and 0.67 for *PPDKs*. Contrastingly, those with higher expression in the KC exhibited larger D‐values for homeologous genes within subgenome A, such as weights_B/AB_ of 0.31 for *NAD‐MEs* and 0.29 for RuBisCO small subunits (Figure 2g). These results indicate cellular subgenome dominance, where subgenome B preferentially contributes to the C_4_ steps in IMC, whereas subgenome A preferentially contributes to the C_4_ steps in KC. This provides new insights into the coordination and evolution of homeologous elements in allotetraploid C_4_ plants.

### 2.6 Single‐Nucleus *cis*‐Regulatory Atlas for C_3_ and C_4_ Culms

**Figure 3:**
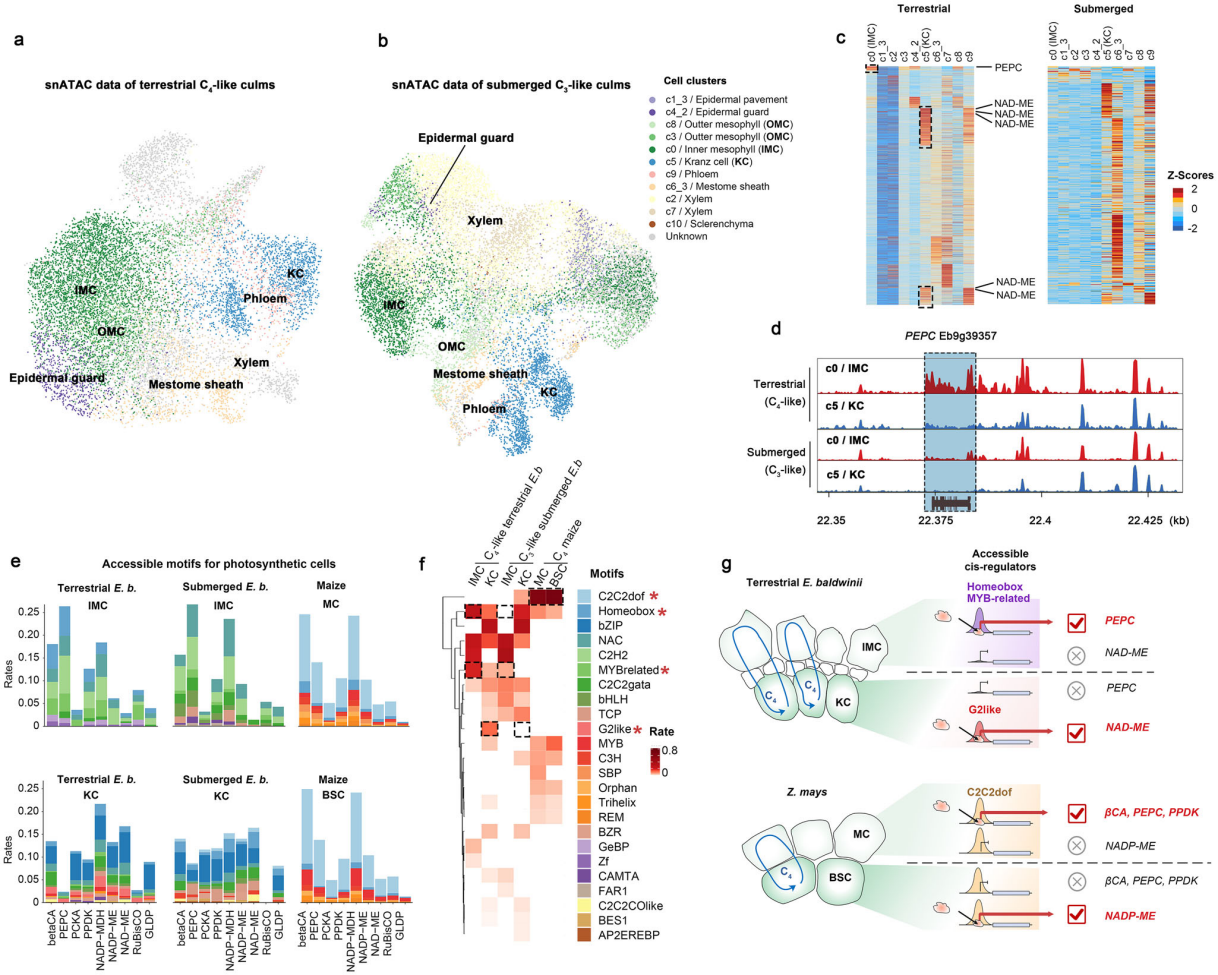
Single nucleus ATAC‐seq reveals *cis*‐regulatory elements for the C_4_ genes. a,b) The Uniform Manifold Approximation and Projection (UMAP) plots of snATAC data showed the cell clusters of terrestrial (a) and submerged (b) culms, annotated by matching into the snRNA‐seq profiles. c) The heat maps represented the chromatin accessibility of cell‐specific expressional genes. The dashed frames indicate the IMC‐ and KC‐specific ones. d) The peaks of snATAC‐seq reads along *PEPC* illustrate the cell‐specific chromatin accessibility. e) The bar plots represent the frequency of the accessible *cis*‐regulatory elements along each gene category. Sum of the rates of element frequency in the genes per each cell type of the specimens (one separated panel) was 1. f) The heat map represents the frequency of accessible *cis*‐regulatory elements for all C_4_ genes per each cell type of the specimens (one separated panel deposited in (e)). In (f), the asterisks represent the key regulatory elements for the C_4_ genes. Large cellular distinctions between terrestrial and submerged *E. baldwinii* or high levels in maize are highlighted by dashed frames. Sum of rates of element frequency in each cell type (column) is 1, represented by the color scale. g) The model indicates that the monocots selected different *cis*‐regulators for establishing the C_4_ pathway.

We performed transposase‐accessible chromatin sequencing in single nuclei (snATAC‐seq) to assess the cellular regulatory mechanism of gene expression. For the 12‐day‐after‐transplantation culms, we obtained 12801 nuclei (biological replicates, n = 6) under terrestrial conditions and 11800 nuclei under submerged conditions (n = 3), with an average of 9536 fragments in each cell. We then aggregated the biological replicates and assigned cell types (**Figure** **3**a,b, Figure S12a,b, Supporting Information). Our findings showed high matching possibilities (0.8‐0.95) for KC (c5) nuclei between snATAC‐seq and snRNA‐seq, and a relatively lower score (0.4‐0.5) for IMC (c0) (Figure S12c, Supporting Information). This aligns well with the c5 distribution (KC) in the snRNA Uniform Manifold Approximation and Projection (UMAP) plots, showing a clear division from other clusters (Figure 2b), whereas c0 (IMC) was dispersed without distinct boundaries. This indicates that KC demonstrates lower cell heterogeneity than IMC. Furthermore, marker genes in snRNA‐seq datasets show chromatin accessibility in each cell type (Figure 3c); and the peaks of snATAC reads were detected around a marker gene *PEPC* at IMC of terrestrial C_4_‐like culms (Figure 3d). These results confirm the accuracy of snATAC‐seq cell‐type assignments, especially IMC, providing confidence in identifying cell‐specific cis‐regulatory elements in these peaks. Additionally, we validated these assignments via *de novo* clustering of the snATAC‐seq nucleus, and the resulting clusters closely resembled those assigned by snRNA‐seq as expected (Figure S12d,e, Supporting Information).

### 2.7 Regulatory Elements for C_4_ Metabolism in *E. baldwinii*

Next, we investigated cell‐specific cis‐regulatory elements in core C_4_ genes (Figure 3e), based on reliable snATAC read peaks (Tables S9 and S10, Supporting Information). We generated a heat map (Figure 3f) based on the average motif rates of all C_4_ genes to facilitate clear comparison across cell types in various environments. We observed that distinctions in motif accessibility between the IMC and KC remained dramatic compared to the distinctions between terrestrial and submerged environments. In both environments, the NAC (average proportion = 24.2% in IMC and 9.9% in KC) and C2H2 (28%, 0%) families dominated all C_4_ genes in IMC, and the bZIP (0%, 34.8%) family was dominant in KC (Figure 3f). Subsequently, we identified high‐rate motifs under terrestrial compared to submerged conditions as core regulators for C_4_‐like forms; we found Homeobox (21.6%, 0%) and MYB‐related (18%, 5%) motifs in IMC, whereas G2‐like motifs (10.4%, 0%) in KC (highlighted by dashed frames and asterisks in Figure 3f). This complex *cis*‐regulatory pattern of the C_4_ gene suggests a complex photosynthetic regulation in *E. baldwinii* via cell specialization and environmental responses.

### 2.8 Distinct C_4_ Gene *cis*‐Regulators Among Monocots

By combining single‐cell ATAC‐seq datasets from maize leaves,^[^ ^43^ ^]^ we evaluated the *cis*‐regulatory motifs of the C_4_ genes (Figure 3e), which were largely distinct from those in *E. baldwinii*. In the heatmap displaying average motif frequencies across all C_4_ genes, the C2C2dof motifs dominated in both MC and BSC types of maize (averaging 63.3% prevalence), but environment‐induced motifs in *E. baldwinii* (e.g., Homeobox, MYB‐related, and G2‐like) showed limited utilization in these cells of maize (the four mentioned motifs were indicated by dashed frames and asterisks in Figure 3f). Moreover, C2C2dof remained similar proportions between MC (60%) and BSC (66.5%) in maize, which was constructed with *E. baldwinii* whereas the predominant motif families differed in IMC and KC (Figure 3g). These findings indicated the diverse *cis*‐regulatory patterns for C_4_ genes among monocots; C_4_ formation was linked to cell specialization and environmental responses in *E. baldwinii*, while such links appear to be weaker in maize.

We then tested whether the interspecies distinction of *cis*‐regulatory elements was derived from intrinsic sequence variance or chromatin accessibility. We drew a full picture of the intrinsic promoter sequence composition of the C_4_ genes, *PEPCs* and RuBisCO small subunits, using Poaceae and Cyperaceae genome sequence data (Figure S13, Supporting Information). The motif compositions were similar in the same plant family but varied between families. The AP2/EREBP motifs dominated these genes in Poaceae, whereas the C2C2dof motifs were enriched in Cyperaceae based on genome sequence data, contrasting with the identified accessible *cis*‐regulator data where C2C2dof motifs were enriched in Poaceae. This suggests that the selective access of core regulatory regions beyond the evolution of intrinsic promoter sequences may largely contribute to the formation of *cis*‐regulatory elements.

### 2.9 C_4_ Photosynthesis Involves a Few of the Environmentally Triggered *cis*‐Regulatory Elements

**Figure 4:**
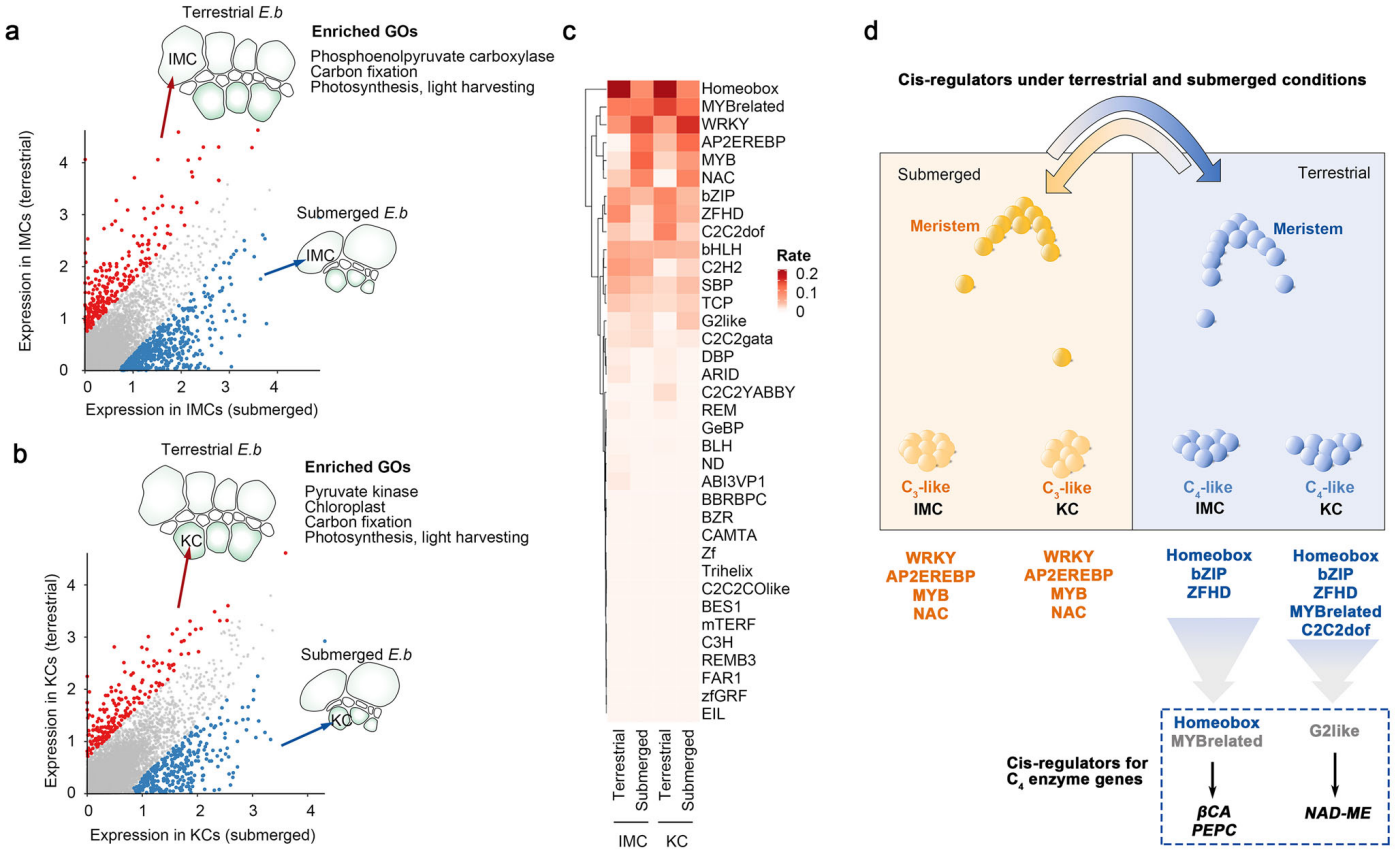
The *cis*‐regulators for terrestrial and submerged growth. a) The dot plot reveals the differently expressed genes between the terrestrial and submerged inner mesophyll cells (IMC). b) The dot plot reveals the differently expressed genes in Kranz cells (KC). In (a) and (b), the red and blue dots represent the highly expressed genes in the terrestrial and submerged cells, respectively. c) The heat map represents the frequency of accessible *cis*‐regulatory elements for differently expressed genes between the terrestrial and submerged cells, deposited in (a) and (b). Sum of rates of the element frequency in the gene set (column) was 1, represented by the color scale. d) The model indicates that the major *cis*‐regulatory elements for terrestrial growth differ from those for submerged growth and reveals limited overlap with the elements governing the genes of the C_4_ enzymes.

Next, we estimated the *cis*‐regulatory linkage between the C_4_ genes and other environment‐differentially expressed genes, to examine the elemental origins. The differentially expressed genes between environmental conditions for each cell type were profiled (see the Methods section for details). In the IMC, 260 and 546 highly expressed genes were found under terrestrial and submerged (**Figure** **4**a; and Table S11, Supporting Information); in KC, 194 and 328 highly expressed genes, respectively, were found (Figure 4b; and Table S12, Supporting Information). Notably, the highly expressed genes in the terrestrial IMC (260) and KC (194) were enriched in gene ontology (GO) terms associated with carbon fixation pathways, like *carbon fixation* (GO:00 15977), and *photosynthesis/light harvesting* (GO:0 009765) enriched in both IMC and KC, as well as *chloroplast* (GO:0 009507) in only KC. Conversely, submerged cells showed no enrichment for their highly expressed genes (546 in IMC; 328 in KC) in these pathways. As expected, the highly expressed genes in the terrestrial cells (260 for IMC and 194 for KC) also included C_4_ enzyme‐encoding genes.

We detected cell‐specific environmentally responsive *cis*‐regulators based on these highly expressed genes (Figure 4c; and Tables S13 and S14, Supporting Information). In these findings, different cell types in the same environment demonstrated similar cis‐regulatory patterns with enrichment of homeobox, bZIP, and ZFHD motifs in terrestrial IMC and KC, and dominant WRKY, AP2/EREBP, MYB, NAC, and G2‐like motifs in submerged IMC and KC. The small distinctions include the fact that MYB‐related and C2C2dof motifs were only found in the terrestrial IMC but not in the KC (Figure 4c). These findings suggest the existence of a *cis*‐regulatory switching system with inducibility of the environment.

A limited overlap was observed when these regulatory elements were compared with those of the C_4_ genes (Figure 4d). In IMC, only the Homeobox motif family was dominant in the C_4_ genes and highly expressed genes under terrestrial conditions. The other predominant families for C_4_ regulation, such as MYB‐related in IMC and G2‐like in KC, existed but did not dominate in regulating those terrestrial‐highly expressed genes (Figure 4d). This suggests that C_4_ metabolism establishment in *E. baldwinii* selectively incorporates motifs from environmentally inducible *cis*‐regulators, which must not belong to the predominant family.

### 2.10 The C_4_ Pathway Selectively Incorporates Non‐Dominant Cell‐Specialized Motifs

**Figure 5:**
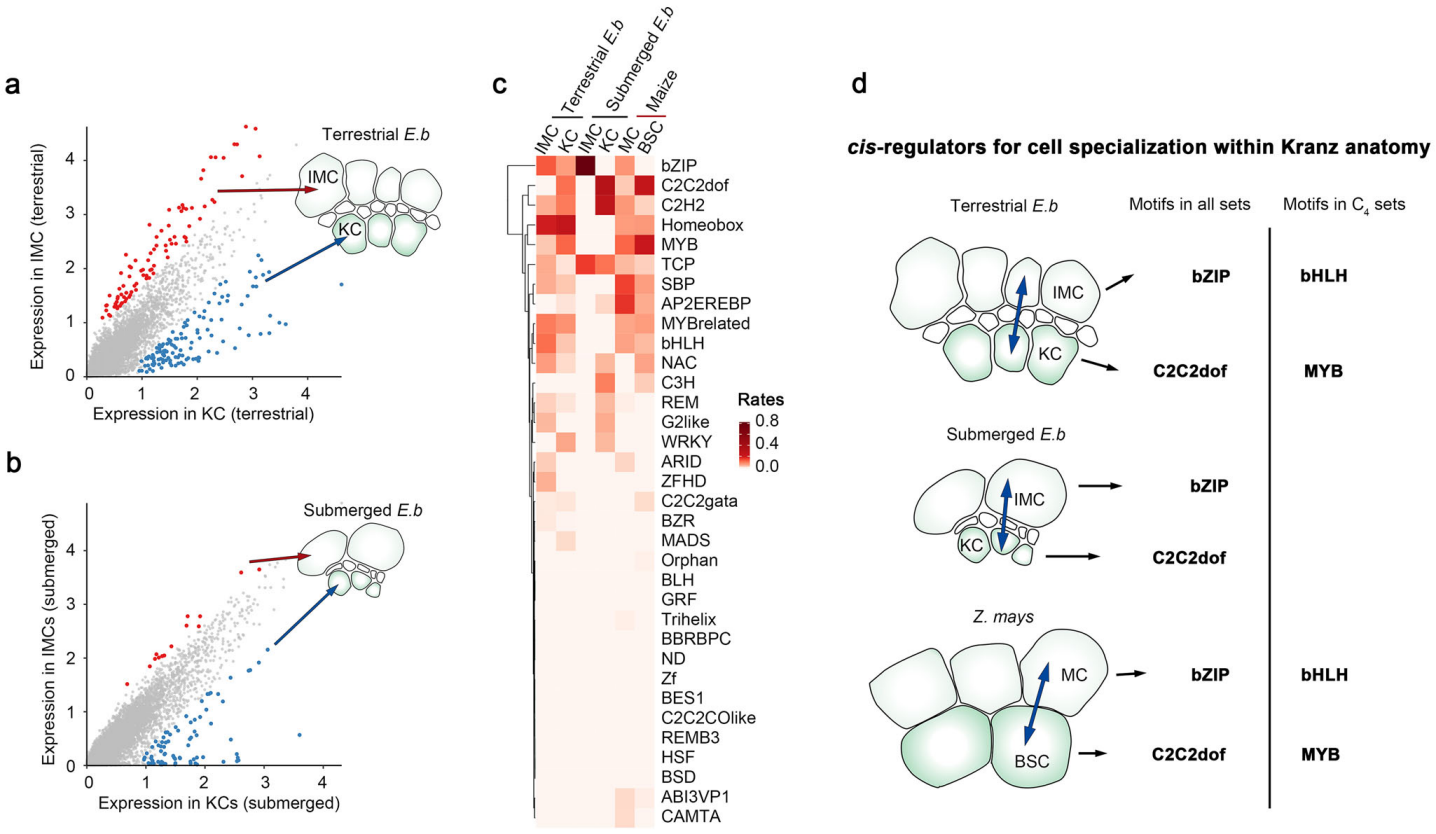
The *cis*‐regulators for cell specialization within Kranz anatomy. a) The dot plots indicate the differently expressed genes between inner mesophyll cells (IMC) and Kranz cells (KC) of the terrestrial culms. b) The dot plots reveal the differently expressed genes between IMC and KC of the submerged culms. The red and blue dots indicate the genes highly expressed in IMC and KC, respectively. c) The heat map represents the frequency of accessible *cis*‐regulatory elements for the IMC‐ or KC‐specialized genes under both conditions, deposited in (a) and (b). Sum of the rates of the element frequency in the gene set (column) was 1, represented by the color scale. d) The diagram reveals the *cis*‐regulatory elements for whole‐cell specialized genes across different plants.

Performance of the C_4_ carbon fixation pathway depends on the cell‐specialized distribution (IMC and KC) of core enzymes within the Kranz anatomy. We assessed the *cis*‐regulatory linkage of C_4_ genes and other differentially expressed genes between IMC and KC (see Experimental Section for details) to assess the impact of cell‐specialized regulation on C_4_ pathway establishment. Comparing the IMC and KC of terrestrial C_4_‐like forms, 87 highly expressed genes in IMC and 125 in KC were identified (**Figure** **5**a; and Table S15, Supporting Information). Furthermore, 14 highly expressed genes in IMC and 79 in KC were found in the submerged C_3_‐like forms (Figure 5b; and Table S15, Supporting Information). The *cis*‐regulatory elements of cell‐specialized genes were subsequently identified using snATAC‐seq read peaks (Figure 5c; and Tables S16 and S17, Supporting Information). Quantitative comparisons confirmed cell‐specialized motifs predominant in both C_4_‐like and C_3_‐like forms: bZIP preferentially in IMC (C_4_‐like: 13.6% vs KC 7.0%; C_3_‐like: 83.9% vs KC 0%) and C2C2dof in KC (C_4_‐like: 11.4% vs IMC 0%; C_3_‐like: 31.4% vs IMC 0%). Beyond these, we identified cell‐specialized motifs exclusively in C_4_‐like forms: bHLH preferentially in IMC (11.5% vs KC 3.4%) and MYB in KC (12.3% vs IMC 3.3%). Given that these predominant motif families were distinct from the dominant motifs of the C_4_ genes (Figure 3e,f), we propose that the C_4_ pathway in *E. baldwinii* selectively incorporates motifs from cell‐specialized *cis*‐regulators within the Kranz anatomy, beyond *cis*‐regulator repertoires in response to environments (see above Section 2.9.; Figure 4d).

### 2.11 Asymmetric Regulation of Gene Expression between OMC and IMC

Most C_4_ species such as maize perform C_4_ photosynthesis within a single mesophyll layer and its adjacent BSC. Physiologically, longer diffusion distances would hinder the efficient transfer of four‐carbon compounds to BSCs if the distal mesophyll layers are involved in C_4_ photosynthesis, leading to decreased efficiency. Next, we investigated differences in gene expression and *cis*‐regulatory patterns between OMC and IMC to explore how IMC, but not OMC, participates in the C_4_ pathway. We identified a limited distinction between OMC and IMC at the gene expression level: only 26 differently expressed genes in the terrestrial C_4_‐like form and 25 in the submerged C_3_‐like form were present, with over half of the genes linked to photosynthesis (14 genes in C_4_‐like and 15 in C_3_‐like forms), including *PEPCs*, RuBisCO small subunits, and photosystem ii subunits (Table S18, Supporting Information). The higher expressions of photosynthesis‐associated genes in OMC than in IMC, suggests that the participation of IMC in the C_4_ pathway may be relied on IMC‐specific inhibition of these carbon fixation genes. We identified *cis*‐regulatory elements of these genes using snATAC‐seq read peaks. A limited number of motifs were detected, including a bZIP motif enriched in these IMC lower expressed genes under terrestrial condition (Table S19, Supporting Information). Considering that bZIP *cis*‐regulators dominated in terrestrially induced genes (Figure 4c,d) and IMC‐specialized genes (Figure 5c,d), this finding supports that bZIP family may play a key role in construction of C_4_ steps within IMC.

### 2.12 Conserved Regulatory Patterns for Kranz Specialization in Different Plants

We further investigated *cis*‐regulatory patterns of Kranz specialization and assessed their contributions to C_4_ metabolism in different plants. We analyzed publicly accessible datasets of single‐cell transcriptomes of leaves from two representative species: maize (C_4_)^[^ ^44^ ^]^ and rice (C_3_).^[^ ^45^ ^]^ The gene expression levels of each cell type were profiled after cell clustering and annotation (Figure S14 and Table S20, Supporting Information). We identified differentially expressed genes between MC and BSC: 237 highly expressed genes in MC and 228 in BSC in maize, and 320 highly expressed genes in MC and 330 in BSC in rice (Tables S21 and S22, Supporting Information). On screening highly expressed genes exclusively from C_4_ forms (terrestrial *E. baldwinii* and maize) but from C_3_ forms (submerged *E. baldwinii* and rice), we detected only 20 C_4_‐conserved genes (eleven for IMC; nine for KC) (Figure S15, Supporting Information), including C_4_ enzyme genes such as *CA2* (*Eb9g39239*, *Zm00001d011454*), *RBCS1A* (*Eb9g40901*, *Zm00001d052595*), *GLDP* (*Eb10g07254*, *Zm00001d023437*; *Eb9g38943*, *Zm00001d023437*), and *RCA* (*Eb10g04485*, *Zm00001d048593*). These findings suggest that cell‐specialized genes may be less cooperative among the different C_4_ plants.

We further identified *cis*‐regulatory elements for these MC (IMC)‐ and BSC (KC)‐ specialized genes in maize (Figure 5c; and Table S23, Supporting Information). Similar predominant families of *cis*‐regulatory elements were observed within the C_3_‐ and C_4_‐like forms of *E. baldwinii*, including bZIP motifs in MC (IMC) and C2C2dof motifs in BSC (KC). However, some predominant motif families overlapped with those in C_4_‐like forms but not in C_3_‐like forms in *E. baldwinii*, such as the bHLH motifs in MC (IMC) and MYB motifs in BSC (KC) (Figure 5d). Although *E. baldwinii* and maize diverged ≈94 Mya, our findings highlight a conversed regulatory mechanism for cell specialization within the Kranz anatomy in monocots, especially in the C_4_ forms. Additionally, compared to the motif families for regulating C_4_ genes (Figure 3e,f), these results revealed a similar predominant *cis*‐regulator family for C_4_ genes and other cell‐specialized genes in maize, including C2C2dof motifs. However, the predominant families differed in regulating the two gene sets of *E. baldwinii*. These findings suggest that C_4_ metabolism establishment in maize largely employs *cis*‐regulatory elements for cell specialization within the Kranz anatomy, whereas that in *E. baldwinii* selectively incorporates motifs from broader repertoires for environmental responses and cell specialization. C_4_ photosynthesis in different plant lineages demonstrates varying selection ratios for elements derived from various regulatory processes.

## 3 Discussion

### 3.1 Molecular Basis for the Plastic C_3_‐C_4_ Photosynthesis in *E. baldwinii*

**Figure 6:**
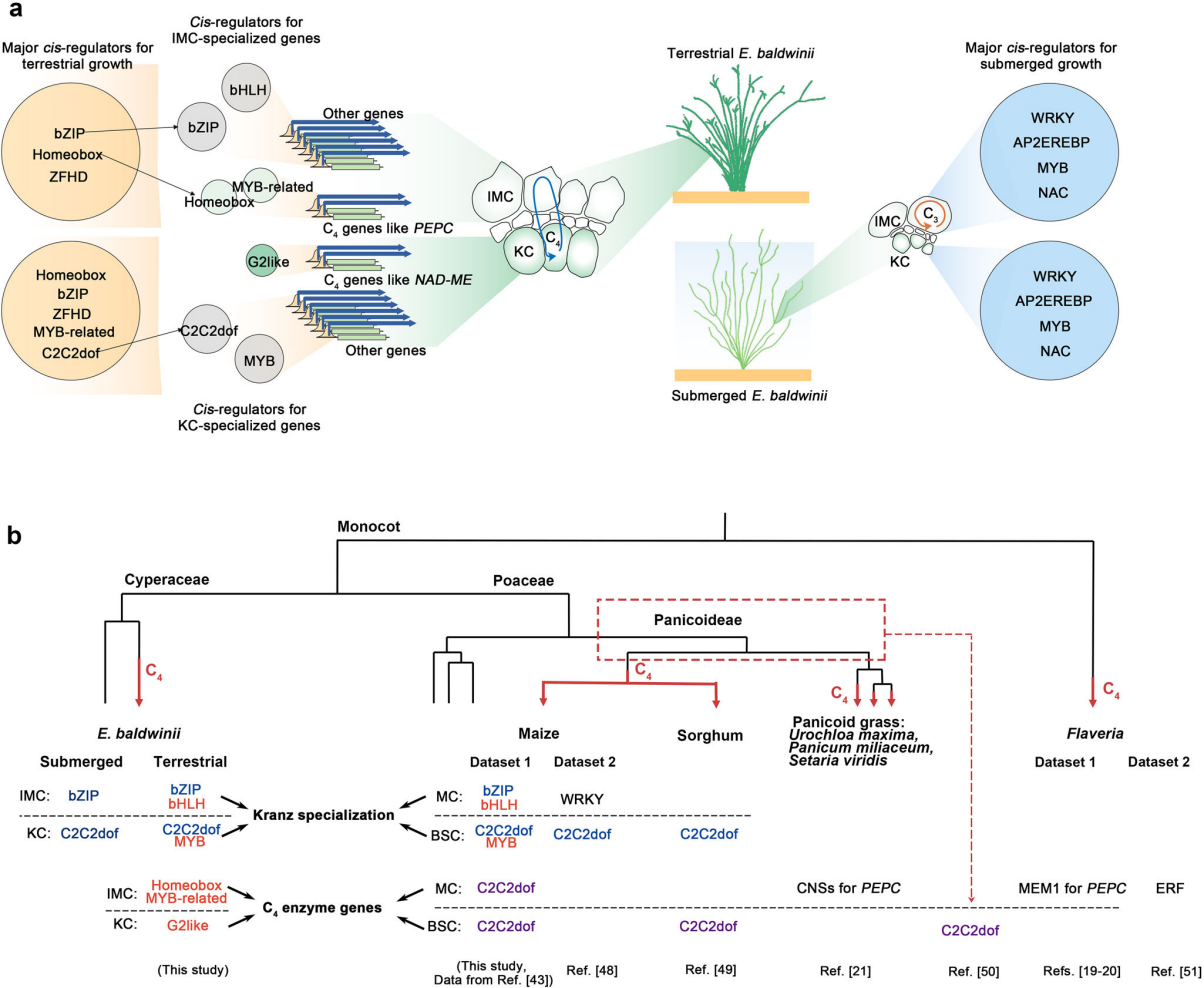
The selection of *cis*‐regulators for C_4_ photosynthesis across plants. a) A summary view of the predominant *cis*‐regulatory elements for C_4_ gene expression, cell specialization, and growth under various environments. The *cis*‐regulatory elements for C_4_ gene expression reveal limited overlap with those predominant elements for cell specialization and terrestrial growth. b) An exhibition of *cis*‐regulatory elements for C_4_ genes and whole cell‐specialized genes, detected in this study and collected from publications.

This study explored the molecular connections between environmental factors and C_4_ photosynthesis establishment, by using *E. baldwinii* exhibiting an environment‐inducible photosynthetic plasticity.^[^ ^28^, ^29^ ^]^ We observed that the C_3_ or C_4_ photosynthetic pathways remained stable in mature *E. baldwinii* culms, and alternate environmental conditions switched the developmental fates of the meristem into C_3_‐ or C_4_‐like forms (Figure 1g). Assembly of the complete allopolyploid genome of *E. baldwinii* allowed us to explore the evolution of gene block regions containing C_4_ genes. C_4_‐specific *PEPCs* were not colinear across Poaceae and Cyperaceae, suggesting that non‐colinear copies of enzyme genes independently evolved for neofunctionalization to establish the C_4_ pathways. We applied single‐nucleus RNA‐seq to C_3_‐ or C_4_‐like culms for profiling the cellular expression pattern of C_4_ genes. We found that homeologous genes between subgenomes do not equally contribute to C_4_ photosynthesis. Subgenome B genes prefer C_4_ steps within the IMC and subgenome A prefers steps within the KC, which we termed cellular subgenome dominance. This provides new insights into the genomic evolution of allotetraploid C_4_ plants (Figure 2g). Single‐nucleus ATAC‐seq allowed us to systematically assess *cis*‐regulatory patterns linked to the C_4_ pathway, Kranz anatomy specialization, and environmental responses. Our results showed that the formation of the C_4_ pathway involves a few environmental‐induced, cell‐specialized *cis*‐regulatory elements (Figure 4d), including Homeobox, MYB‐related in IMC, and G2‐like in KC. However, not all these motifs belong to the predominant families for regulating environmental responses and Kranz anatomy specialization. This suggests that C_4_ photosynthesis selectively incorporates *cis*‐regulators from a broader repertoire shaped by environment alterations and anatomy development in *E. baldwinii* (**Figure** **6**a).

### 3.2 Adaptation Evolution of Photosynthetic Plasticity

Our results provide new insights into the evolution of photosynthetic plasticity in *E. baldwinii*, especially with respect to historical climate change. Plasticity, with thicker C_4_‐like culms under terrestrial conditions and thinner C_3_‐like culms under submerged conditions, appears to be an effective adaptation to rapidly changing water levels in swamp habitats. Submerged culms were unable to stand upright when removed from water, whereas terrestrial culms, which developed thicker KC and vascular tissues, demonstrated normal upright growth. Larger KC in terrestrial culms may increase mechanical support and water transport while also creating conditions favorable for the C_4_ pathway development in terrestrial environments. High temperatures and dry conditions further promote stomatal closure of terrestrial culms, decreasing water transpiration, and consequently lowering mesophyll‐interstitial CO_2_ levels. C_4_ pathway establishment in terrestrial culms may further increase CO_2_ fixation and enhance plant adaptability to these habitats, especially during periods of low atmospheric CO_2_. However, the natural selection of thin culms under submerged conditions implies their benefit for submerged growth, which may decrease water drag in fluid mechanics.^[^ ^46^, ^47^ ^]^ Our findings revealed that the C_4_ pathway and cell specialization within the Kranz anatomy incorporated environment‐switching *cis*‐regulators (Figure 6a). This suggests a subtle designation for coordinating anatomical and metabolic regulation perhaps in adaptation to rapidly changing water levels. This study provides a glimpse of the molecular connections of how historical environments shaped the emergence of C_4_ traits.

### 3.3 *Cis*‐Regulator Evolution of C_4_ Photosynthesis Across Different Plant Lineages

We collected *cis*‐regulatory elements of C_4_ genes and other cell‐specialized genes across species to systematically summarize the genetic basis of convergent C_4_ photosynthesis^[^ ^19^, ^20^, ^21^, ^43^, ^48^, ^49^, ^50^, ^51^ ^]^ (Figure 6b). Although the predominant *cis*‐regulators of MC‐ or BSC‐ specialized genes remain relatively conserved, the selection of *cis*‐regulatory elements for C_4_ genes distinctly differs among various lineages. The results from our comparative analyses show that *E. baldwinii* uses environmentally switched *cis*‐regulators to govern C_4_ gene expression but maize dose not. The previous study shows that another C_4_ grass, sorghum, appeared to favor cell‐identity networks to enable C_4_ photosynthesis.^[^ ^49^ ^]^ The molecular difference corresponds with their respective habitats: unlike E. baldwinii which experiences fluctuating water levels, maize and sorghum predominantly grow in relatively stable conditions. These findings highlight how different evolutionary strategies in cis‐regulatory elements have enabled C4 photosynthesis adaptation to diverse ecological niches.

Additionally, *cis*‐regulatory elements for C_4_ genes tend to be relatively conserved within the same family, and several studies have reported that C2C2dof elements are widely used in C_4_ species of the Poaceae family.^[^ ^49^, ^50^ ^]^ Similarly, in *Flaveria*, a progressive evolution of key regulatory elements such as ERFs has been observed during the transition from C_3_ to the C_3_‐ C_4_ intermediate and finally to C_4_ photosynthesis.^[^ ^51^ ^]^ These results suggest that while various C_4_ plant lineages may use distinct regulatory elements to initiate C_4_ pathways, species within the same lineage likely recruit relatively similar regulatory pathways, possibly due to more conserved genetic foundations.

Moreover, we observed that the whole promoter sequences of C_4_ genes, detected in genome sequence data, are largely different from the identified accessible *cis*‐regulators. This implies that selective access of core regulatory regions beyond the evolution of intrinsic promoter sequences may primarily contribute to the formation of *cis*‐regulatory elements. Thus, our data highlights a significance of identifying cellular *cis*‐regulatory elements in assessing convergent C_4_ photosynthesis evolution. This study provides genomic and regulatory evidence for understanding how plants have evolved C_4_ photosynthesis, especially regarding the impact of environmental factors.

## 4 Experimental Section

### Plant Materials and Genome Sequencing

*E. baldwinii* individuals used in this study were planted at 28 °C with 16 h light/8 h dark in the Institute of Genetics and Developmental Biology, Chinese Academy of Science, Beijing, China. Mature culms were harvested for DNA extraction of short reads and HiFi read sequencing using the Illumina Hiseq‐3000 platform (150 bp paired‐end) conducted by BGI (Shenzhen, China) and the PacBio Sequel System conducted by Berry Genomics (Beijing, China), respectively. The same individual used for HiFi long‐read sequencing was utilized for Hi‐C library construction and sequencing with the Illumina NovaSeq6000 platform (150 bp paired‐end) conducted by Berry Genomics (Beijing, China).

### Endogenous ABA Measurement

Approximately 200 mg (fresh weight) of the plant tissue was homogenized in liquid nitrogen, weighed, and extracted with methanol and 2H6‐ABA for 24 h. Endogenous ABA was purified and measured as previously described^[^ ^53^, ^54^ ^]^ with modifications to the detection conditions. The LC‐MS/MS analysis was conducted using an ultra‐performance liquid chromatography system (Waters) coupled with a 6500 QTrap system (AB SCIEX). LC separation was performed on a BEH C18 column (1.7 µm, 100 × 2.1 mm; Waters) using 0.05% (v/v) acetic acid in water as mobile phase A and 0.05% (v/v) acetic acid in acetonitrile as mobile phase B. The gradient was initially set at 20% B and subsequently increased to 70% B within 6 min. ABA was detected in the multiple reaction monitoring (MRM) mode with transition. The MRM transitions for ABA and [2H6]‐ABA were 263.0>153.1 and 269.2>159.2, respectively. Three biological replicates were analyzed for each treatment.

### Measurement of PEPC and RuBisCO Enzyme Activities

Fresh culms of 12‐day‐after‐transplantation of terrestrial, ABA‐treated, and submerged *E. baldwinii*, along with rice and maize leaves, were collected and weighed. The PEPC and RuBisCO enzyme activities were separately quantified using 0.1 g samples, and the experimental procedures were conducted according to the instructions provided in the reagent kit (G0606W; G0602W48, Suzhou Grace Biotechnology Co., Ltd, Suzhou, China). The experiments were repeated at least six times.

### Genome Size and Heterozygosity Estimation

Raw short reads of *E. baldwinii* were processed using fastp^[^ ^55^ ^]^ (v0.20.1) to remove poor‐quality base calls and adaptors. Jellyfish^[^ ^56^ ^]^ (v2.2.10) was used to evaluate the distribution of 31‐mer frequency based on the trimmed short reads. Smudeplot^[^ ^57^ ^]^ (v0.2.3dev) was subsequently used to extract heterozygous k‐mer pairs and estimate ploidy. Finally, GenomeScope2^[^ ^57^ ^]^ (v2.0; p4) was used to estimate *E. baldwinii*’s genome size and heterozygosity.

### Flow Cytometry

*E. baldwinii*’s genome size was also estimated via flow cytometry^[^ ^58^ ^]^ using Nipponbare and maize varieties B73 as an internal standard. Terrestrial *E. baldwinii* was placed in a 500‐µL nuclei lysis buffer (15 mm Tris‐HCl pH 7.5, 20 mm NaCl, 80 mm KCl, 0.5 mm Spermine, and 0.2% Trixton X‐100),^[^ ^59^ ^]^ chopped with a sharp blade, and subsequently filtered using a 40‐µm cell strainer. For each sample, 10000 cells were collected by adding a final concentration of 50 µg mL^−1^ staining buffer with RNase and propidium iodide for 30 min in the dark. The DNA peak ratio was evaluated using flow cytometry after 30 min of incubation.

### *De novo* Genome Assembly and Hi‐C Scaffolding

HiFi reads were assembled with Hifiasm ^[^ ^60^ ^]^ (v0.13‐r308) using default parameters. Clean Hi‐C reads were subsequently mapped to primary assembled contigs using BWA‐ALN^[^ ^61^ ^]^ (v0.7.17). The assembly errors were corrected and the contigs were ordered using SALSA^[^ ^62^, ^63^ ^]^ (v2.3) pipeline. Before mapping to the primary assembled contigs, the raw reads generated from the Hi‐C library were trimmed using fastp to remove adaptors and low‐quality reads. For gap closure, the HiFi reads were first assembled using Canu^[^ ^64^ ^]^ (v2.1.1) and PB‐assembly^[^ ^65^ ^]^ (v0.0.2). Next, the contigs generated with HiCanu and PB‐assembly (which were called adjusted contigs here) were mapped to the above scaffolds using minimap2^[^ ^66^ ^]^ (v2.20). Finally, if their flanking sequences were well aligned with the scaffolds, the gaps were replaced by the corresponding sequences in the adjusted contigs. Short reads from whole‐genome resequencing were mapped to primary contigs using BWA‐MEM^[^ ^61^ ^]^ (v0.7.17) to estimate the accuracy of the assembled genome, and genetic variance (SNPs and InDels) was identified using GATK4 HaplotypeCaller (v4.1.6.0; –minimum‐mapping‐quality 30 –min‐base‐quality‐score 20; https://github.com/broadinstitute/gatk). Furthermore, BUSCO was used to estimate the completeness of the assembled genomes. Additionally, the LAI was calculated to evaluate the assembly quality with LTR_retriever^[^ ^67^ ^]^ (v2.9.0) using the output of LTRharvest^[^ ^68^ ^]^ (v1.1).

### RNA Sequencing and Data Analysis

The mRNA of the mature culms, roots, and stems in terrestrial and submerged environments was separately extracted using a Quick RNA isolation kit (Huayueyang, Beijing, China). The mRNA from the above tissues and environments was mixed to approximate contents and sequenced with the PacBio Sequel System to obtain full‐length transcripts conducted by BGI (Shenzhen, China). Subsequently, the IsoSeq3 (https://github.com/PacificBiosciences/IsoSeq) pipeline was applied to obtain high‐quality full‐length transcripts. Circular consensus sequences (v6.0.0) were obtained using the following parameters: min‐length 300 –max‐length 15 000 –min‐rq 0.8 –min‐passes 1 –skip polish. Additionally, the mRNA from mature culms was used to perform RNA‐seq with DNBSEQ‐T7 (150 bp paired‐end), which was conducted by BGI (Shenzhen, China). Raw reads that passed quality control with Trimmomatics^[^ ^69^ ^]^ (v0.36) were mapped to the *E. baldwinii* genome using Tophat2^[^ ^70^ ^]^ (v2.1.1), and transcripts were assembled using Cufflinks^[^ ^71^ ^]^ (v2.2.1). *De novo* assembly of the RNA‐seq was performed using Trinity^[^ ^72^ ^]^ (v2.8.4) under the default parameters.

### Gene Structure and Repeat Annotation

For annotation of protein‐coding genes, First a database was built of *E. baldwinii* using RepeatModeler^[^ ^73^ ^]^ (v2.0.1) and masked the repetitive sequences using RepeatMasker (v4.1.2‐p1, http://repeatmasker.org/RepeatMasker/) under the library constructed above. Subsequently, evidence from ab initio prediction, homologous protein sequences, and transcriptomes was obtained. For ab initio prediction, AUGUSTUS^[^ ^74^ ^]^ (v3.4.0) and SNAP^[^ ^75^ ^]^ (version 2006‐07‐28) were applied under a self‐trained model. First the protein sequences of *Zea mays*, *Setaria italica*, *Oryza sativa*, *Brachypodium distachyon*, *Arabidopsis thaliana*, *Ananas comosus*, and *Musa acuminata* were downloaded from Ensembl Genomes (release‐51) for protein homology evidence. GenomeThreader^[^ ^76^ ^]^ (v1.7.3) was subsequently used to infer *E. baldwinii*’s genome structure based on its homologous protein sequence. For transcriptome evidence, the PASA pipeline^[^ ^77^ ^]^ (v2.4.1) was used to generate comprehensive transcripts by integrating transcripts assembled using Cufflinks and Trinity high‐quality full‐length transcripts. TransDecoder (v5.5.0; https://github.com/TransDecoder/TransDecoder) was used to identify the candidate coding regions. Finally, to predict the gene structure, all evidence was integrated with EVidenceModeler (EVM)^[^ ^77^ ^]^ (v1.1.1). Based on the reliability of the software, a weight of 10 was assigned to the prediction results of the transdecoder, two to GenomeThreader, two to AUGUSTUS, and one to SNAP in the EVM configuration file. The protein sequences were subjected to eggNOG‐mapper^[^ ^78^ ^]^ (v2.1.4), InterProScan^[^ ^79^ ^]^ (v5.52‐86.0), and KofamKOALA^[^ ^80^ ^]^ (v1.3.0) for gene functional annotation. The *de novo* transposable element (TEs) annotator EDTA^[^ ^81^ ^]^ (v1.9.8) with the parameters “–sensitive 1 –anno 1 –evaluate 1” was used to construct the TEs.

### Divergence Time Estimation

To understand the evolutionary history of *E. baldwinii*, first the hmm model of a pre‐defined 1614 embryophyta single copy genes was downloaded from the BUSCO website (https://busco‐data.ezlab.org/v5/data/lineages/), and subsequently, HMMER^[^ ^82^ ^]^ (v3.3) was applied to search the orthologous genes in *Amborella trichopoda*, *Ananas comosus*, *Juncus effusus*, *Rhynchospora tenuis*, *Rhynchospora pubera*, *Rhynchospora breviuscula*, *Carex littledalei*, *E. vivipara*, *E. baldwinii*, *Streptochaeta angustifolia*, *Pharus latifolius*, *Zizania latifolia*, *Oryza sativa*, *Triticum aestivum*, *Hordeum vulgare*, *Brachypodium distachyon*, *Setaria italica*, *Sorghum bicolor*, and *Zea mays*. For each species, only the best hit with an e‐value > 1e‐10 was retained. MAFFT^[^ ^83^ ^]^ (v7.487) was subsequently used to perform multiple sequence alignment, and trimAl^[^ ^84^ ^]^ (v1.5) was used to remove ambiguous alignment. Finally, 1608 orthologous genes that appeared in at least 15 species were used for the phylogenetic tree construction and dating analysis using IQTREE2^[^ ^85^ ^]^ (v2.3.6; –date‐ci 100). The subgenome of *E. baldwinii* was separated with SubPhaser^[^ ^38^ ^]^ (v1.2.6) using the default parameters. MCscan^[^ ^86^ ^]^ (Python version v1.1.17) was used to search the synteny region using the longest transcript in each protein‐coding gene for the self‐comparison of *E. baldwinii* and the comparison between *E. baldwinii* and *C. littledalei*. Multiple sequence alignment was conducted with MAFFT for pairwise genes in the synteny region, and KaKs_Calculator^[^ ^87^ ^]^ (v2.0) under the YN model was used to calculate synonymous substitution values (Ks). Mutation rates were calculated using the formula *r* = K/2*T*, where K represents the average value of Ks and T represents the divergence time between *E. baldwinii* and *C. littledalei*. In contrast, the divergence time between the sub‐genomes of *E. baldwinii* was calculated using the formula *T* = K/2*r*, where K is the mean value of Ks between the self‐comparisons of *E. baldwinii*.

### Evolutionary History of *PEPC*

MCscan^[^ ^86^ ^]^ (Python version v1.1.17) was used to search the synteny region of the species (as shown in Figure 1j) using the longest transcript in each protein‐coding gene to explore PEPC's evolutionary trajectory.

### C_4_ Enzyme Identification in *E. baldwinii*

109 protein sequences were collected for *AlaAT*, *AspAT*, *NAD‐ME*, *NADP‐MDH*, *NADP‐ME*, phosphoenolpyruvate carboxykinase (*PCKA)*, *PEPC*, *PPDK* and *RuBisCO* from *Z. mays*, *O. sativa*, and *A. thaliana* to better understand the evolution of the C_4_ core enzyme. Second, protein sequences from *E. baldwinii* and *C. littledalei* were mapped to the reference database using BLASTP^[^ ^88^ ^]^ (v2.11.0; ‐evalue 1e‐5) for orthologous findings. Similar to species tree construction, multiple alignments of each gene were performed using MAFFT followed by trimAl, and trees were constructed using IQTREE2.

### Single Nucleus Sequencing: Nuclei Preparation and Library Construction

SnRNA‐seq and snATAC‐seq libraries were prepared from at least three independent biological samples. Each was composed of 12‐day‐after‐transplantation culms of *E. baldwinii* under terrestrial, ABA‐treated, and submerged conditions. The nuclei were isolated as previously described ^[^ ^59^ ^]^ with some modifications. In brief, the fresh culms from several samples were placed on Petri dishes and immediately chopped with a razor blade in ≈1 mL pre‐chilled lysis buffer (15 mm Tris‐HCl pH 7.5, 20 mm NaCl, 80 mm KCl, 0.5 mm Spermine and 0.2% Trixton X‐100, snRNA‐seq required additional 0.5 U µL^−1^ SUPERase RNase inhibitor). Subsequently, the chopped slurry was filtered two times via a 40‐µm cell strainer. For sorting, the crude nuclei were stained with 4,6‐diamidino‐2‐phenylindole (DAPI) and loaded onto a flow cytometer with a 100‐µm nozzle. At least 200000 nuclei were collected based on the DAPI signal and nuclear size. The sorted nuclei were pelleted at 4 °C and 1000 ×*g* for 5 min, and subsequently resuspended in ≈10 µL lysis buffer without TrixtonX‐100. The concentration and quality of nuclei were estimated using a hemocytometer under fluorescence using the DAPI channel and adjusted to ≈1500 and 3000 nuclei per microliter with nuclease‐free water, respectively. The SnRNA‐seq and snATAC‐seq libraries were prepared from a total of ≈15000 and 50000 nuclei per library, respectively. They were then loaded onto the DNBelab C Series platform, where snRNA‐seq libraries used the Single‐Cell Library Prep Set (MGI, 1 000 021 082)^[^ ^89^ ^]^ and snATAC‐seq libraries were prepared using the Single‐Cell ATAC Library Prep Set70 (MGI, 1 000 021 878).^[^ ^90^ ^]^ Libraries were sequenced on a DNBSEQ‐T1, DNBSEQ‐T7, or BGISEQ‐500 sequencer at the China National GeneBank (Shenzhen, China), respectively.

### Reads Mapping and snRNA‐seq Integration

Reads with valid barcodes were aligned to the *E. baldwinii* genome using STAR,^[^ ^91^ ^]^ and the UMI count matrix of each sample was generated using PISA^[^ ^92^ ^]^ (v1.10.2), with only uniquely mapped reads utilized. Only cells with UMIs > 500 and genes >200 were retained for the following analysis to remove low‐quality cells. The batch effects of samples under different environments were removed using the Seurat R package^[^ ^92^, ^93^ ^]^ (v4.1.1). The gene expression levels were normalized using “SCTransform”.^[^ ^94^ ^]^ Subsequently, the 3000 most variable genes in all the sequenced samples were selected with “SelectIntegrationFeatures” functions. Finally, “aqABA_Rep3” was set as the reference to integrate all samples.

### Cell Clustering and Cell Type Annotation

Cells were clustered using the function “FindNeighbors” in the Seurat R package to create a K‐nearest neighbors graph starting with the normalized and integrated expression matrix. Subsequently, “FindClusters” was conducted to group cells using a 0.5 resolution, resulting in 11 major clusters. For cell types (c1, c4, and c6) that had a lower proportion (< 50%) of expressed marker genes, these clusters were re‐clustered into sub‐clusters. These clusters and subclusters were dimensionally reduced and visualized for UMAP. Genes of known cell types were collected from *Arabidopsis*, maize, and rice (Refs. in Table S6, Supporting Information) to identify and annotate cell clusters, and subsequently identified the orthologous genes in *E. baldwinii* using blastp (‐evalue: 1e‐5). Sixteen genes with the highest expression levels in *E. baldwinii* single‐nuclei sequencing were regarded as functional orthologs for each marker gene. Finally, cluster and subcluster identities were determined by analyzing the expression patterns of established cell‐type‐specific marker genes (Figure S8, Supporting Information).

### RNA In Situ Hybridization

Fresh culms of terrestrial, ABA‐treated, and submerged *E. baldwinii* 12‐days after transplantation were fixed in Formalin‐Aceto‐Alcohol solution (50% ethanol, 5% acetic acid, and 3.7% formaldehyde), dehydrated through a graded alcohol series (50, 70, 85, 90, 95, and 100%) and Histo‐Clear series, and subsequently embedded in paraffin (Sigma‐Aldrich, Germany). The embedded samples were sliced into 10‐µm sections and mounted on slides. Specific regions of the marker genes were cloned into the *pEASY*‐Blunt3 cloning vector (TransGen, China), and a Digoxigenin RNA labeling kit (Roche) was used for transcription and labeling in vitro. Images were captured in bright‐field mode using an Olympus BX53 microscope (Japan). Table S24 (Supporting Information) lists the primers used for probe synthesis.

### Immunohistochemistry

The fresh culms material of *E. baldwinii* under terrestrial, 5 µM ABA‐treated, and submerged forms were fixed with 4% (v/v) glutaraldehyde in 0.1 M phosphate buffer (pH 7.2‐7.4) on 4 °C for 3 h and subsequently embedded in 5% agarose. Transverse sections were cut at a 15–20 µm thickness with a vibratome (Leica VT1000 S, Germany) and placed on a 2 mL centrifuge tube. Immunolabeling was performed using the SABC‐(Rabbit lgG)‐POD Kit (SA0021, Solarbio, China) according to the manufacturer's instructions, with minor modifications. Sections were treated with 3% H_2_O_2_ solution for 10 min and subsequently incubated with 5% Bovine Serum Albumin (BSA) in PBST (0.01 M phosphate‐buffered saline, pH 7.2–7.4, containing 0.05% Tween‐20) for 20 min to remove endogenous peroxidase. To detect Rubisco, PEPC, and NAD‐ME, sections were incubated overnight at 4 °C with the following dilutions of primary antibody: 1:250 rabbit anti‐PEPC antibody, which was made by Abmart (Abmart Shanghai Co., Ltd., China) or 1:200 rabbit anti‐Rubisco large subunit (RbcL) antibody (bs‐6988R, Bioss, USA) or 1:300 rabbit NAD‐ME antibody (D163864, BBI, China). Following incubation at 37 °C for 1 h, the sections were washed three times with PBS buffer for 5 min each, and subsequently incubated with horseradish peroxidase‐labeled goat anti‐rabbit secondary antibody (D110058, BBI, China, 1:500 diluted with 1% BSA) at 37 °C for 1 h. The treated sections were stained with 50 µL 20× diaminobenzidine for 5 min, and washed with distilled water to terminate the reaction. The localization of these enzymes in the sections was observed using an Olympus BX53 microscope (Japan).

### GO Enrichment Analysis

GO terms of *E. baldwinii* genes were collected from the functional annotation results using InterProScan (PANTHER database) for all GO enrichment analyses. Enrichment analysis for the given genes was conducted using the clusterProfiler R packages^[^ ^95^ ^]^ (v4.6.0; pvalueCutoff = 0.05, pAdjustMethod = “BH”).

### Differently Expressed Gene Identification in snRNA‐seq

Differentially expressed genes were identified using the “FindMarkers” function with a fold change > 1.5 and an adjusted p‐value < 0.05. These genes were considered highly expressed in specific cell types or photosynthesis pathways (Figures 4a,b and 5a,b) and were analyzed in the context of their respective cell clusters and sample conditions.

### snRNA‐seq Data Analysis of Other Species

Preprocessed expression matrices for leaf snRNA‐seq in *Z. mays* ^[^ ^44^ ^]^ and *O. sativa* ^[^ ^45^ ^]^ were collected. Cell removal, normalization, integration, dimensionality reduction, cluster finding, and annotation were conducted according to the methods described in each study. Differently expressed genes were also identified using the “FindMarkers” function with fold change > 1.5 and *P*‐adjusted value < 0.05 in each species for the cells annotated with MC and BSC. Orthologous genes of maize, rice, and *E. baldwinii* were identified via reciprocal BLAST analysis (maize vs *E. baldwinii* and rice vs *E. baldwinii*), and the e‐value was set as 1e‐10.

### Reads Mapping and snATAC‐seq Integration

Raw sequencing reads were filtered to remove low‐quality bases using PISA, and the filtered reads were aligned to the *E. baldwinii* genome using BWA‐MEM (v0.7.17). BAP2 (v0.6.2; https://github.com/caleblareau/bap) was used to merge barcodes from the same cell and generate read‐accessible fragment files with unique mapped reads for each sample. ArchR^[^ ^96^ ^]^ (v1.0.1) was utilized to filter cells with TSS enrichment scores < 1.5 and fragments < 4000. The Harmony tool^[^ ^97^ ^]^ integrated into ArchR was used to correct for the batch effect.

### snATAC‐seq and snRNA‐seq Integration

The “addGeneIntegrationMatrix” (reducedDims = “Harmony”) function in ArchR was used to calculate the similarity of cells detected by snRNA‐seq data and snATAC‐seq data. Additionally, the origin of cells from terrestrial and submerged environments were separately processed. Cell clusters in each snATAC‐seq cell were transferred from the snRNA‐seq. The “addReproduciblePeakSet” function was used to find the peaks in IMC and KC. The *cis*‐regulated elements for each gene were identified using the “addPeak2GeneLinks” (reducedDims = “Harmony”) functions.

### Statistical Analysis

All experiments were conducted with a minimum of three independent biological replicates. The figure legends provide the details of the Data pre‐processing and sample sizes (n) for each statistical analysis. The error bars in the figures represent the standard deviation (SD) of the mean. Statistical analyses were performed using GraphPad Prism 8. The two‐tailed paired Student's *t*‐test was used to examine significant differences between two groups, whereas the one‐way ANOVA test was used to assess significant differences between more than two groups. Asterisks (∗) indicate statistical significance at *P* < 0.01 and *P* < 0.0001, respectively.

## Conflict of Interest

The authors declare no conflict of interest.

## Author Contributions

L.C., Y.J., and Z.Z. contributed equally to this work. X.L., X.L., and Y.L. designed and supervised this work. Y.J., Z.Z., Q.J., and J.L. completed the physiological experiments and sequencing library constructions. L.C., J.G., and C.C. analyzed the sequencing data. S.C. and J.C. analyzed the ABA content. L.C., Y.J., Xin Liu, Y.L., and X.L. prepared the manuscript. All authors discussed the results and commented on the manuscript.

## Supporting information



Supporting Information

Supplemental Figures S1–S15 & Supplemental Tables S2–S24

## Data Availability

The reference assembly is available at the China National Center for Bioinformation (CNCB) under the accession number PRJCA031286. The raw datasets used for genome survey, assembly, Hi‐C scaffolding, annotations (Iso‐seq and RNA‐seq data), single‐nucleus RNA‐seq, and ATAC‐seq data were deposited into National Center for Biotechnology Information (NCBI) SRA database under the accession numbers PRJNA1056472, PRJNA1056461, PRJNA1057346, and PRJNA1059289.
