# Supplementary material for: Genomic and Cis‐Regulatory Basis of a Plastic C3‐C4 Photosynthesis in Eleocharis Baldwinii
Source: Adv Sci (Weinh). 2025 May 30;12(32):e15681. doi: 10.1002/advs.202415681 (PMC12407342; doi:10.1002/advs.202415681)
Supplement: Supplementary file 1 — Supporting Information [file ADVS-12-e15681-s001.docx]

**
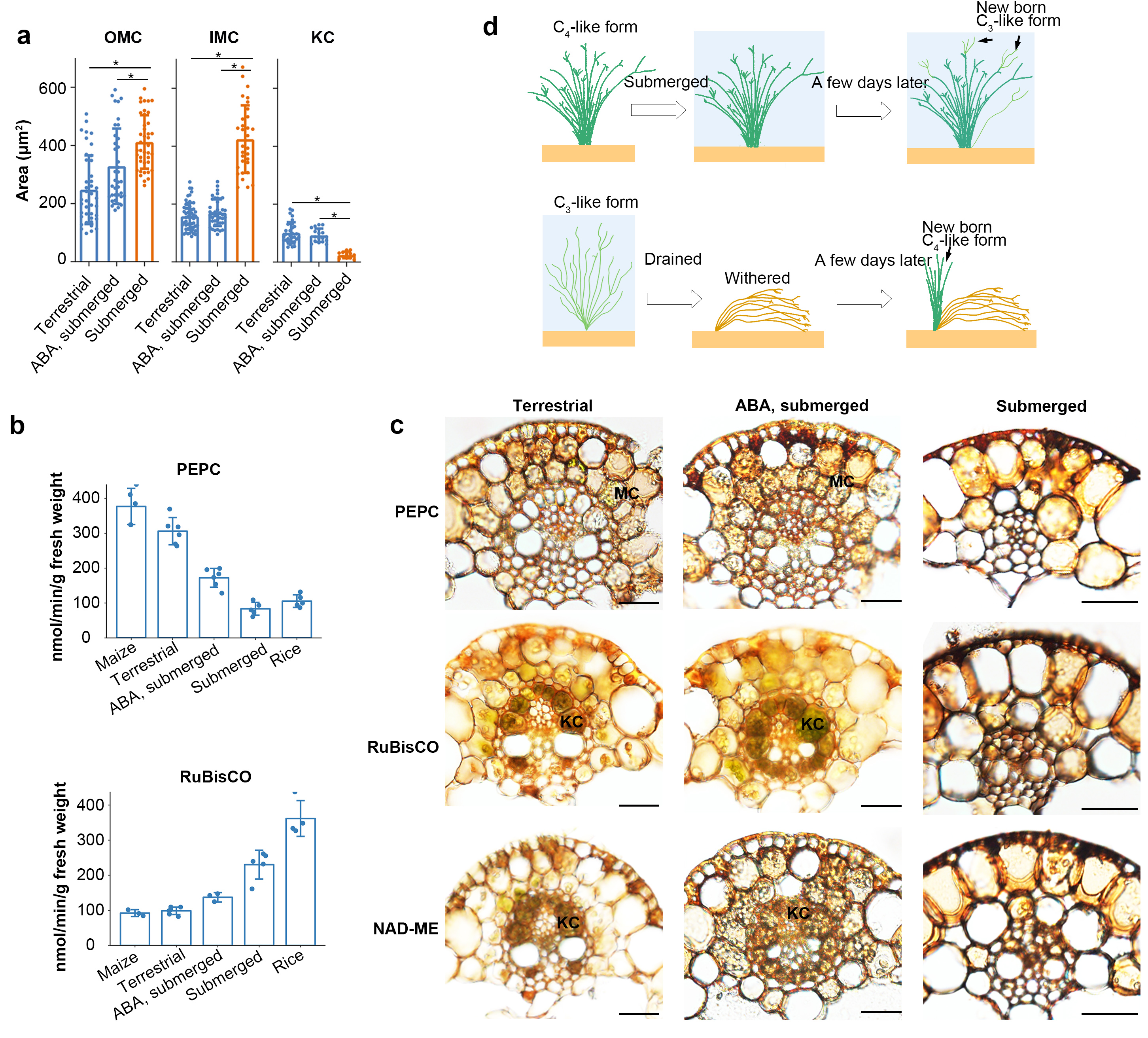
Supplementary figures**

**Figure S1. Phenotyping the photosynthetic plasticity of *E. baldwinii*.**

(a) The area of OMC, IMC, and KC within culms under different environments. Data are presented as means ± SD, n ≥ 12, statistical analyses are performed using one-way ANOVA with Bonferroni correction, * *P* < 0.01. (b) The PEPC and RuBisCO activity of culms in *E. baldwinii*, leaves in maize and rice. The activity was normalized to the tissue fresh weight. See details in the Experimental Section. Data are presented as mean ± SD, n ≥ 3. (c) The immunological detection of C_4_ enzymes. In terrestrial C_4_-like form, RuBisCO and NAD-ME were enriched in KC, while PEPC was enriched in MC. Bar=20 μm. n = 3. (d) The model for switching C_3_ and C_4_ forms. The culms newly born in terrestrial and submerged environments were growing into the C_4_- and C_3_-like forms, respectively, while the mature terrestrial culms maintain its C_4_ pathway behind submerging it. n = 3.


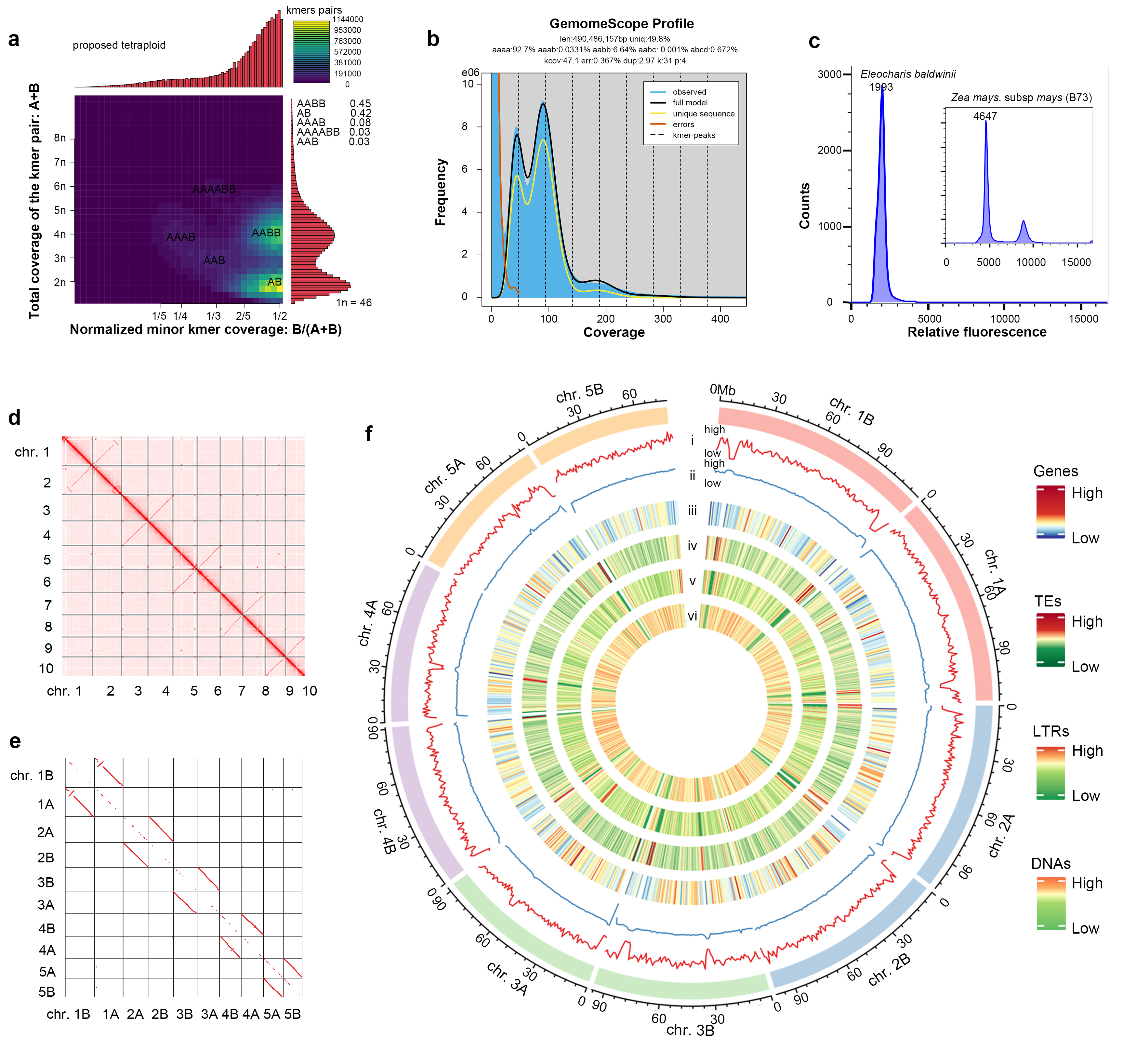


**Figure S2. Genome features of *E. baldwinii.***

(a) Frequency of each kind of haplotype structures that calculated from heterozygous k-mer pairs. The heat of the smudge indicates how frequently the haplotype structure is represented in the genome compared to the other structures. Possibility of ploidy were presented in the right; the estimated ploidy was marked in the top of picture. (b) The 31-mer frequency distribution and estimated genome size, repeat sequence and heterozygous of *E. baldwinii*. The genome size, ploidy, heterozygous rate, repeat sequence level of genome, and the proportion of different kind k-mer pairs were showed in the top of the picture. (c) The nucleus DNA abundance assessed by flow cytometry were shown for *E. baldwinii*. The embedded plot shows those for *Z. mays*. (d) The heat map of Hi-C chromatin interactions. (e) The synteny plot suggesting that *E. baldwinii* genome contains five pairs of homeologous chromosomes. (f) Circus plots of *E. baldwinii* genome. Tracks from outside to inside: ⅰ, LAI score. ⅱ, GC content. The density of gene (ⅲ), all transposon elements (ⅳ), LTRs (ⅴ), DNA transposons (ⅵ).

**
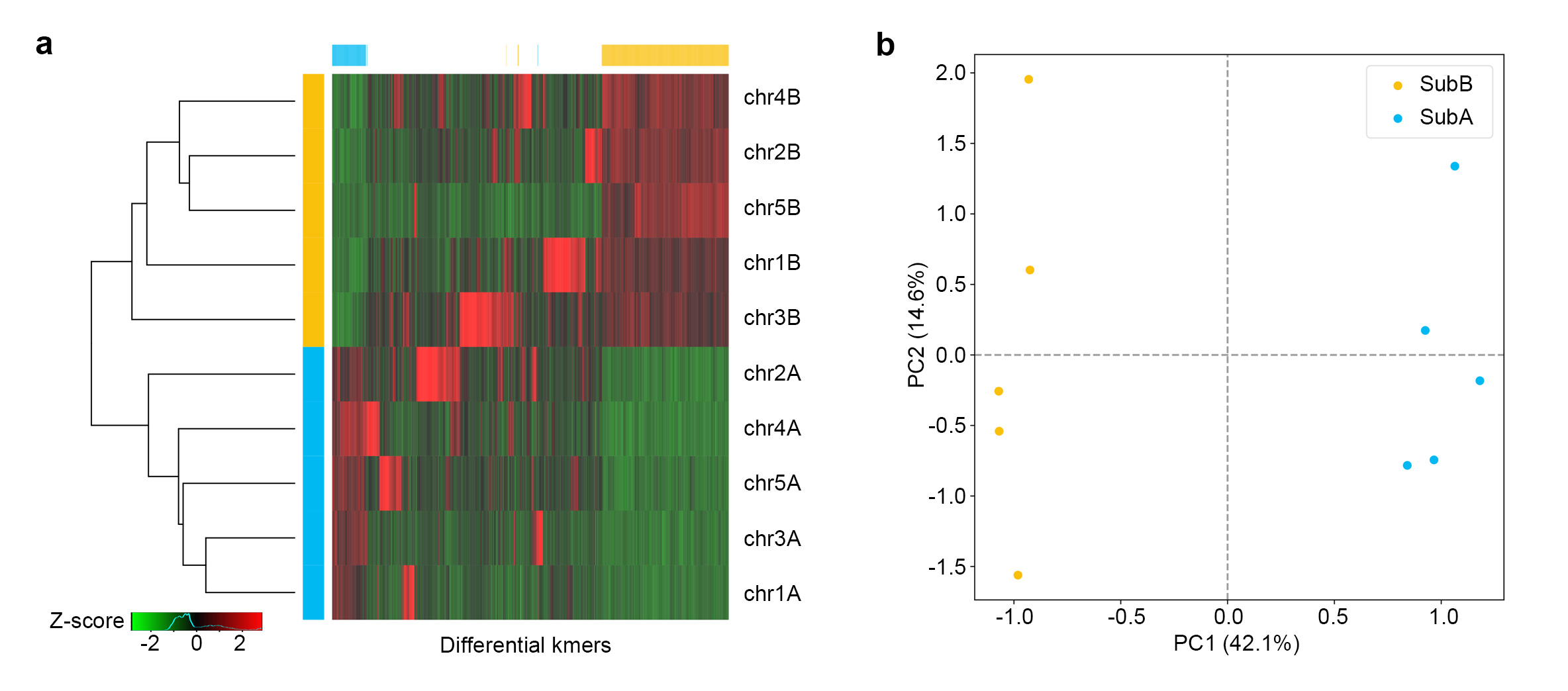
**

**Figure S3. Subgenome separation based on k-mer analysis.**

(a) Heatmap of different k-mers. The horizontal bars show the specific f k-mer to the subgenome, and vertical color bars show the assignment of chromosomes to subgenome. (b) Principal component analysis of differential k-mers from chromosomes.

**
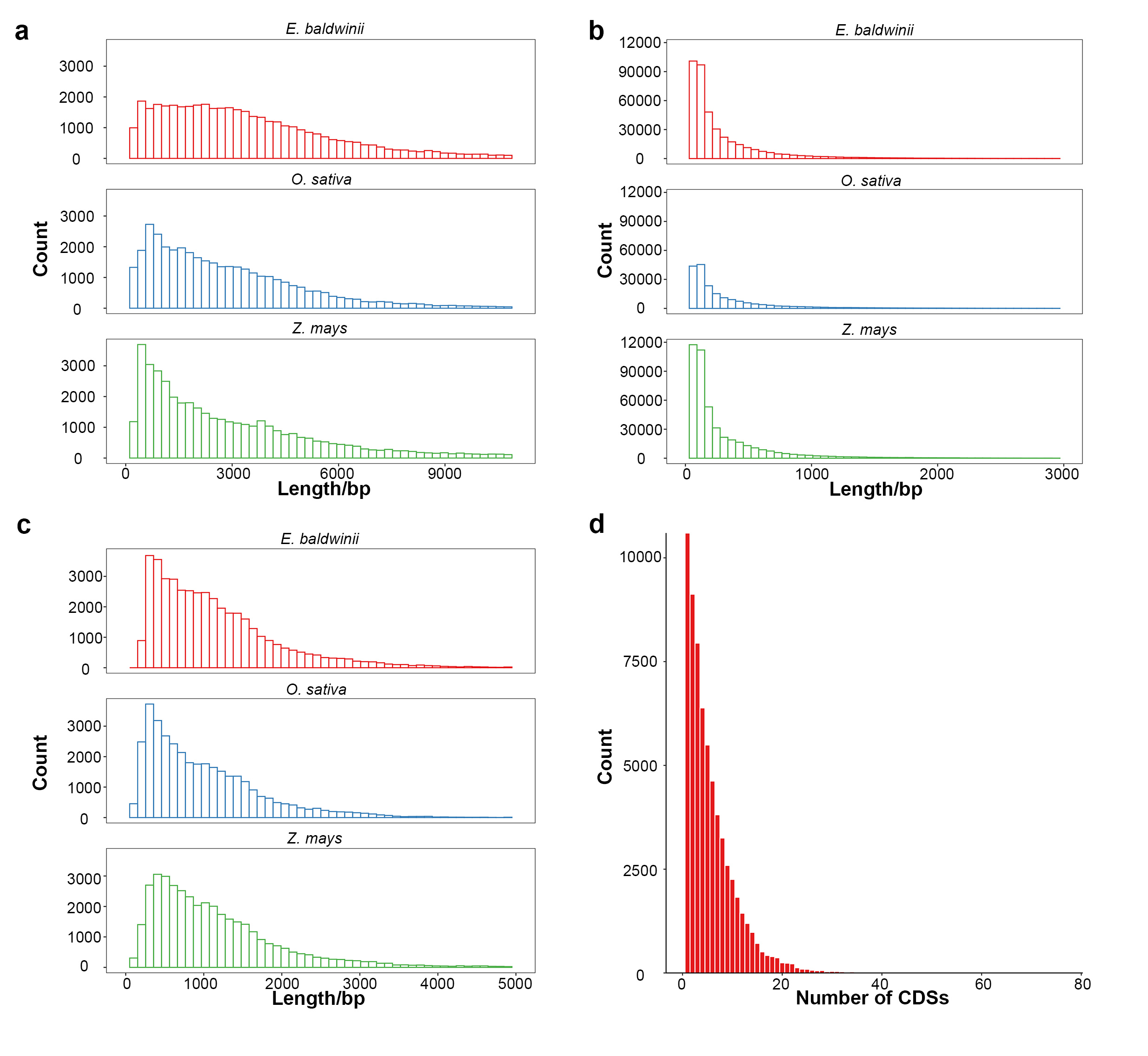
**

**Figure S4. Comparison of gene attributes of *E. baldwinii* to other species.**

(a-c) Length distribution of protein coding genes (a), exon (b), CDS (c). (d) The distribution of CDS numbers.

**
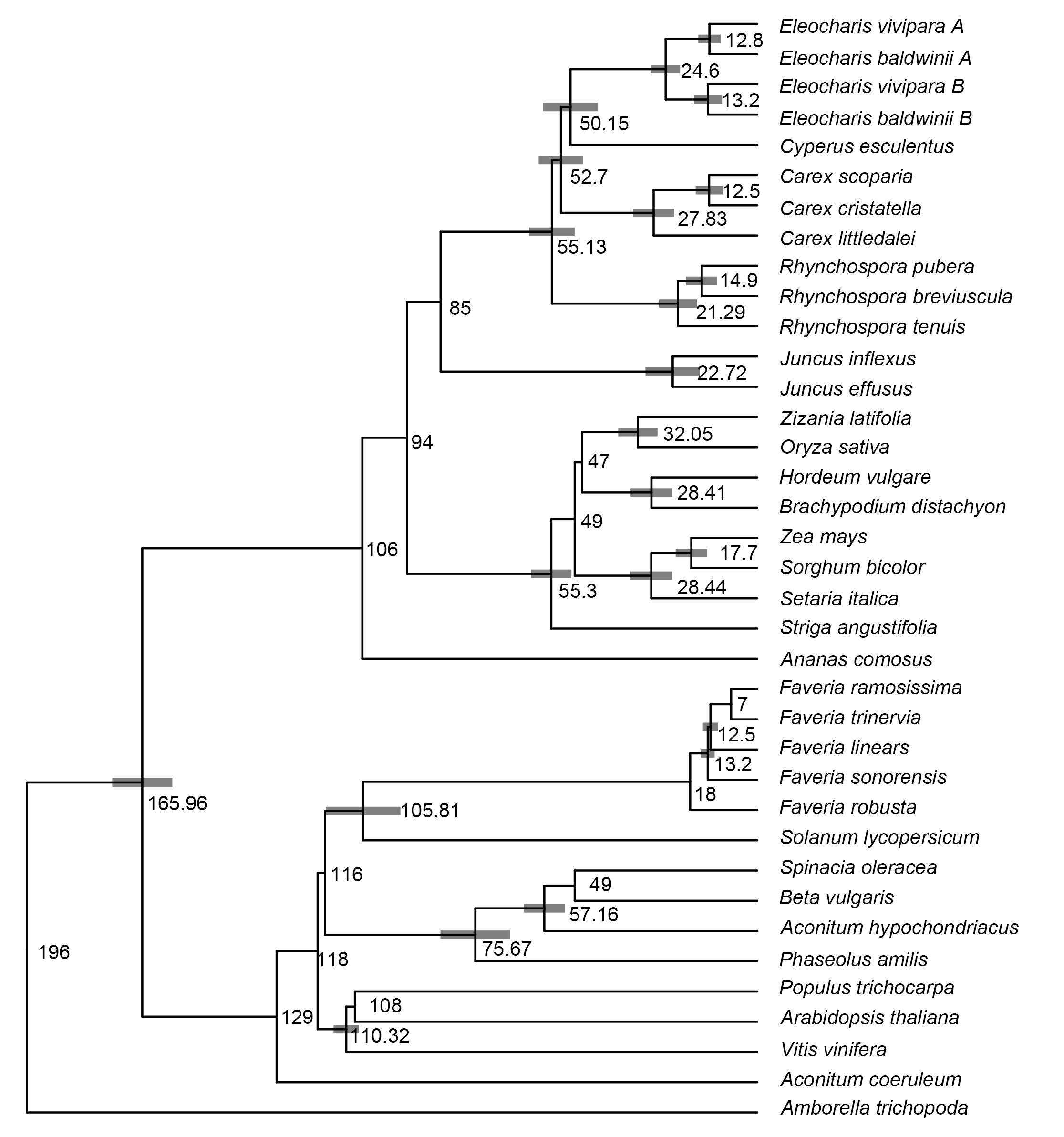
**

**Figure S5. Full** **phylogenetic tree of *E. baldwinii* and other angiosperm species.**

This phylogenetic tree incorporates genome data of Cyperaceae (9 species), Poaceae (8 species), Juncaceae (2 species), Bromeliaceae (1 species), dicots (14 species), and the ancient angiosperm *Amborella trichopoda*, including the distinguished subgenomes of *E. vivipara* and *E. baldwinii*. Bars indicate the estimated confidence intervals.

**
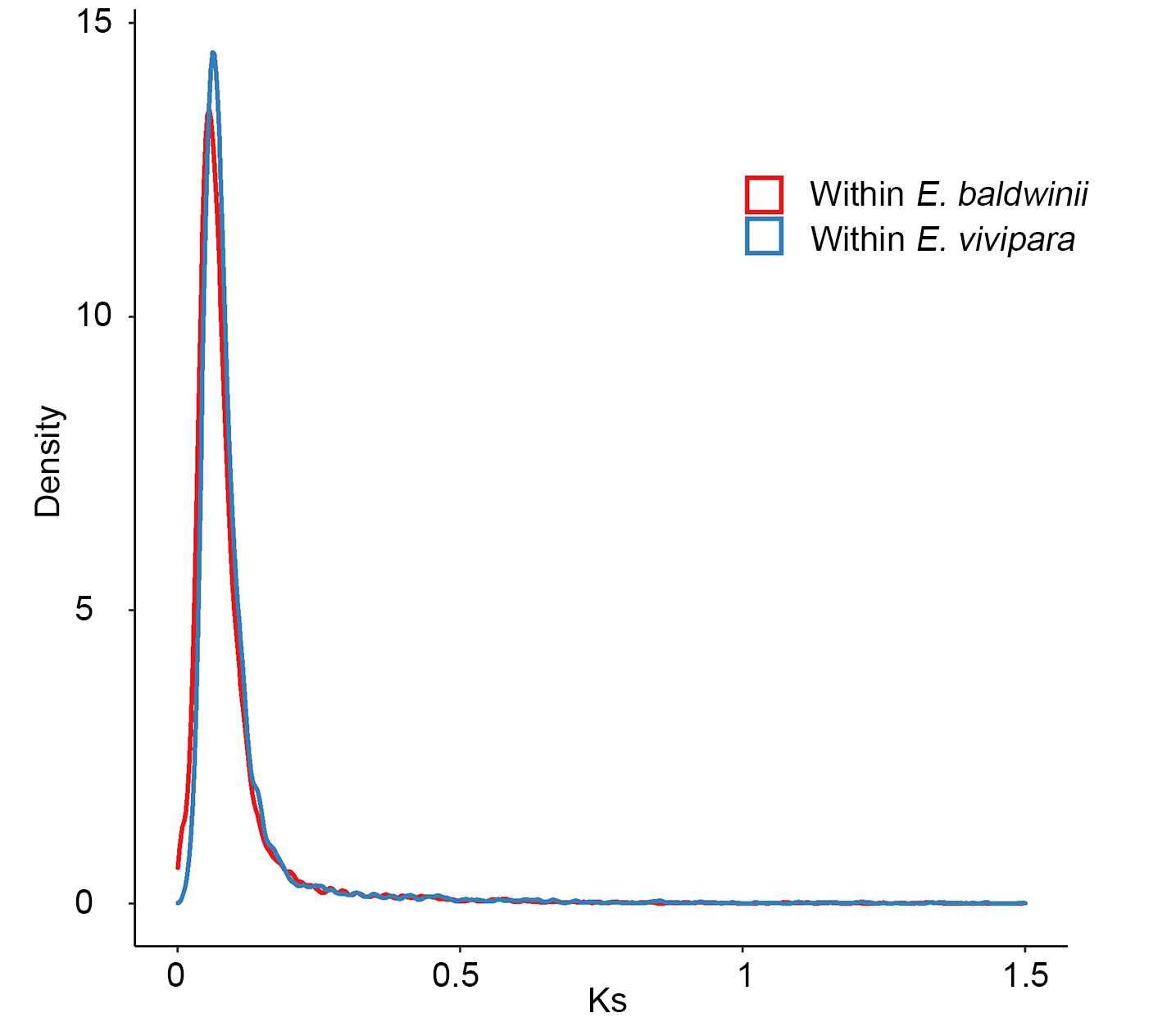
**

**Figure S6. Ks density within *E. baldwinii* and** ***E. vivipara*.**


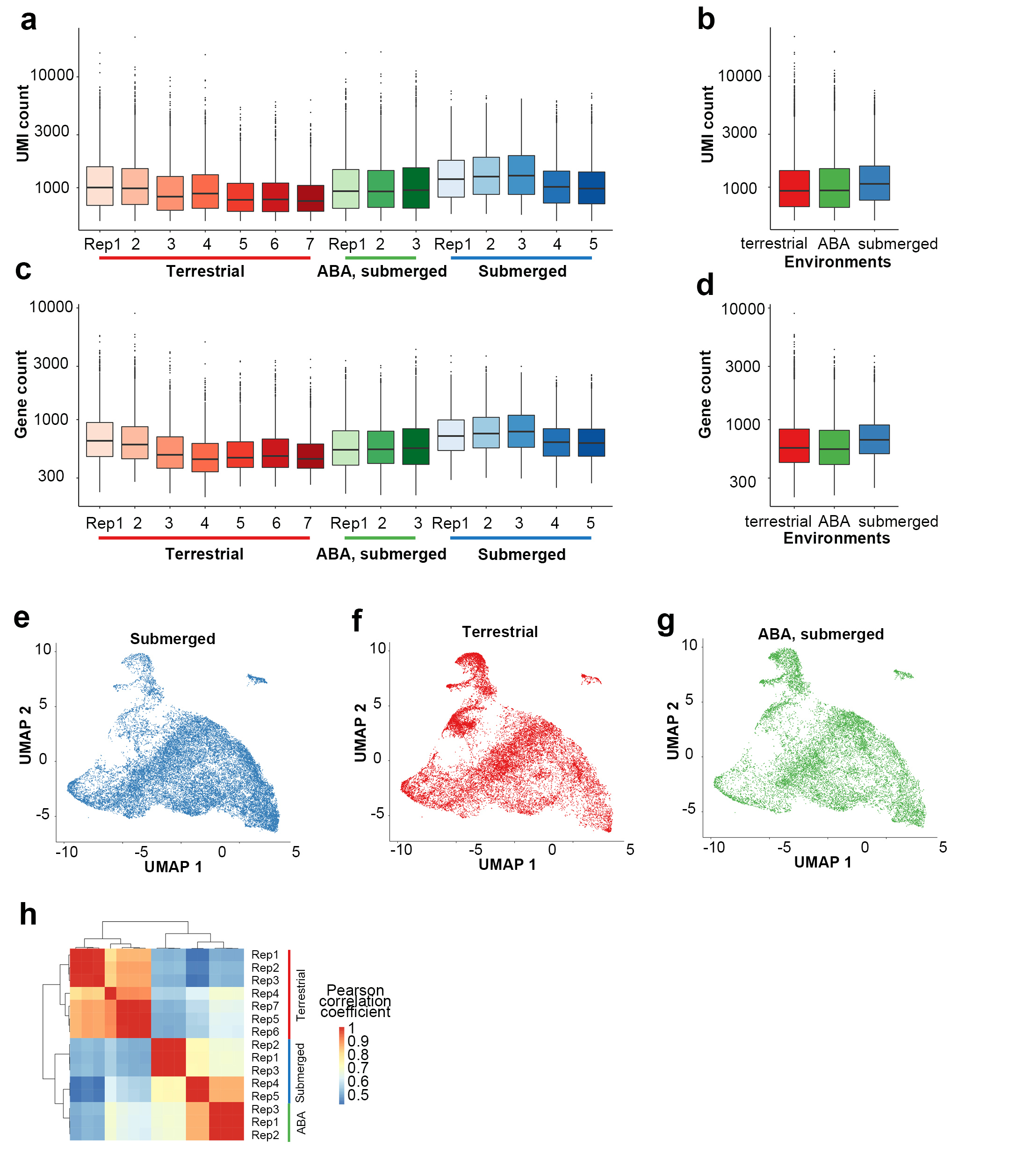


**Figure S7. Quality control of *E. baldwinii* single-nucleus RNA-Seq.**

(a,b) UMI counts of each sample replicates (a) and environments (b). (c,d) Gene counts of each sample replicates (c) and environments (d). e-g UAMP of plot showing two-dimensional representation of cell relationships in multidimensional space for single-nucleus sequencing of mature culms under submerged (e), terrestrial (f) and ABA treated submerged (g) environments. (h) Pearson's correlation coefficients between the snRNA-Seq biological replicates under different environments.

**
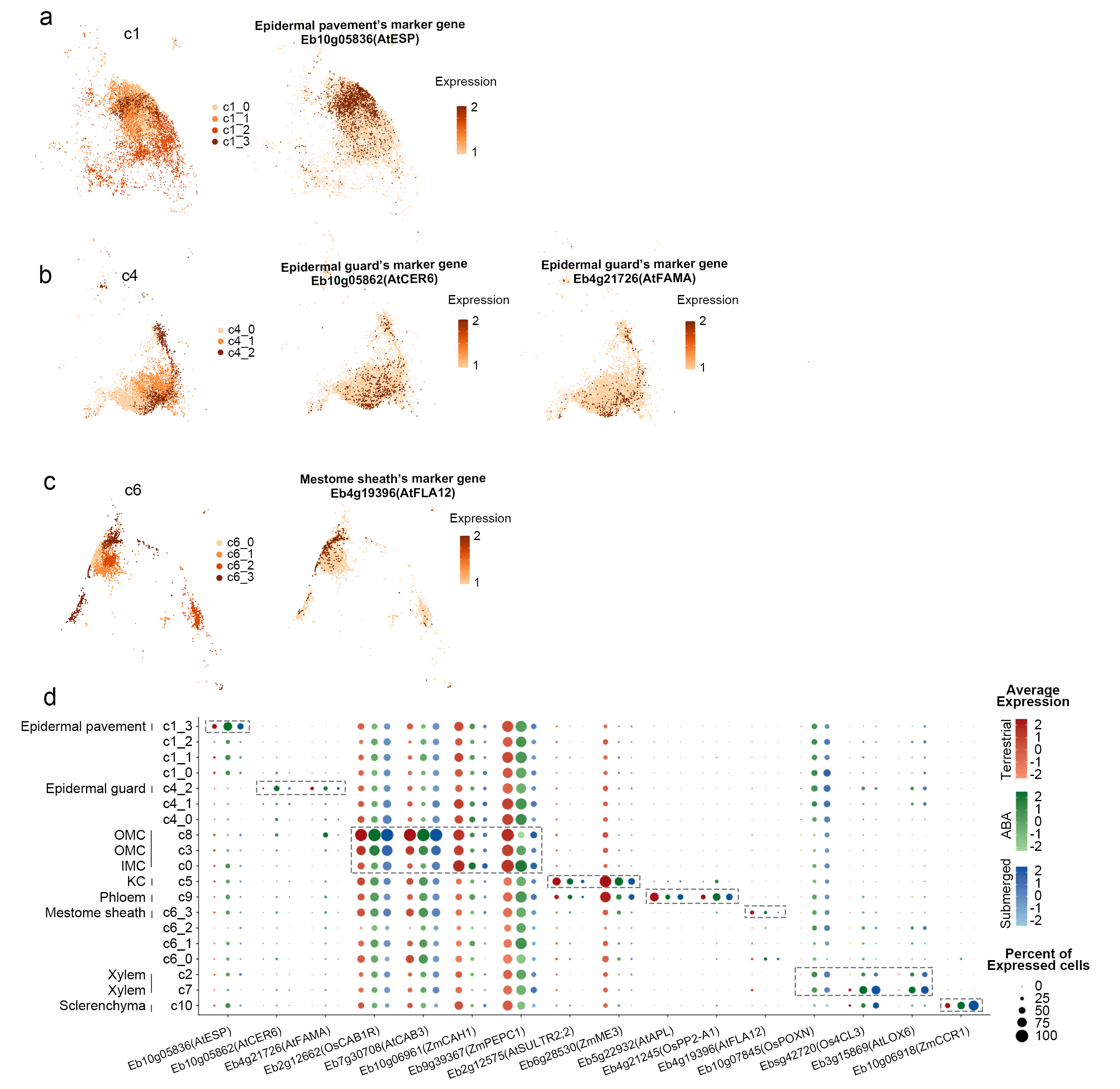
**

**Figure S8. Cell type annotation of *E. baldwinii* single-nucleus RNA-Seq.**

(a-c) The subclusters of c1 (a), c4 (b), and c6 (c). The expression levels of marker genes serve c1_3 as epidermal pavement (a), c4_2 as epidermal guard cells (b), c6_3 as mestome sheath (c). (d) The expressions of 16 known maker genes that defined the major cell types. Colors showed the expression under different environments.

**
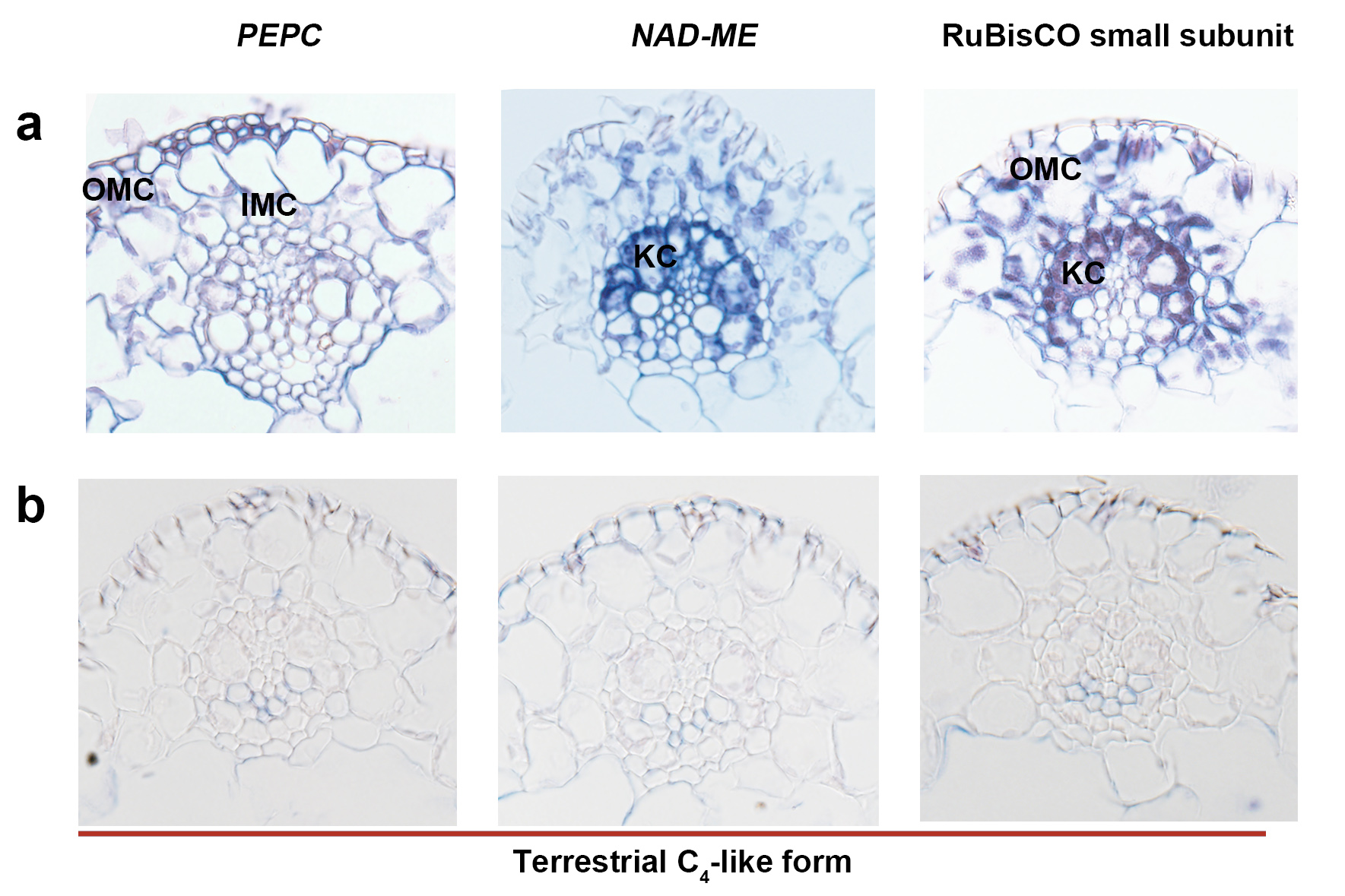
**

**Figure S9. RNA *in situ* hybridization assay for the marker genes.**

(a) RNA *in situ* hybridization assay for each gene in the C4-like culms under terrestrial environments. (b) Blank control for RNA *in situ* hybridization assay. n = 3.

**
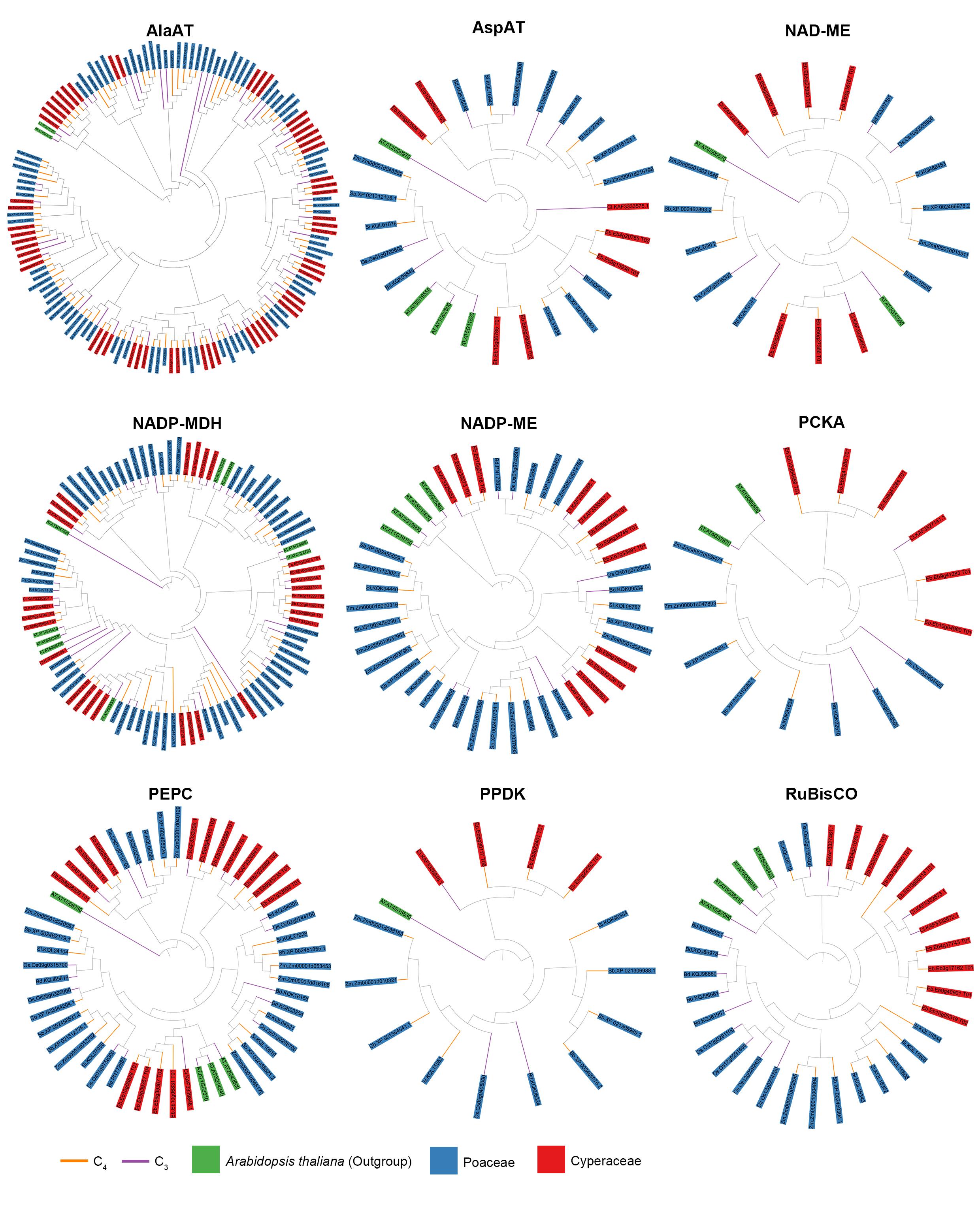
**

**Figure S10. Phylogenetic tree of C_4_ related genes.**

**
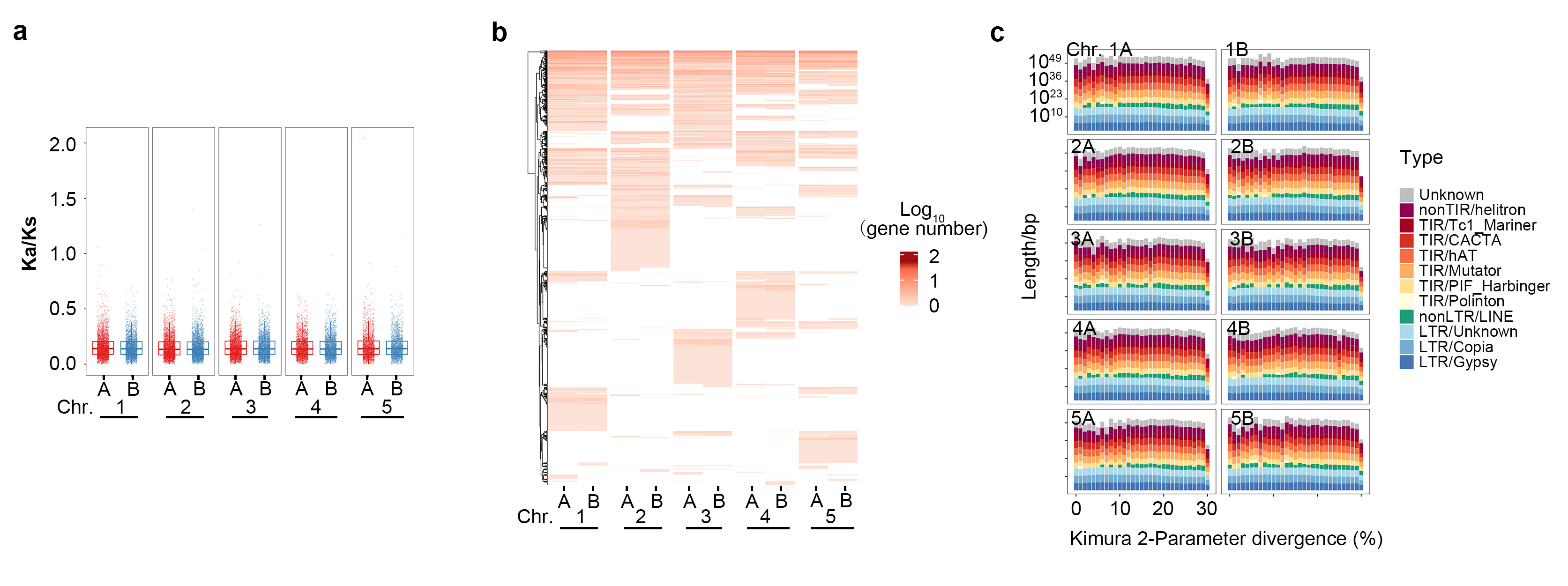
**

**Figure S11.** **Estimation of sub-genome dominance of *E. baldwinii.***

(a) Selective pressure measured with nonsynonymous mutation and synonymous mutation rate in 10 chromosomes of *E. baldwinii*. The five homologous chromosome pairs were shown. (b) Heat map showed the number of genes in each gene families in 10 chromosomes of *E. baldwinii*. Each row represented a gene family. (c) Transposable elements distribution across *E. baldwinii*.


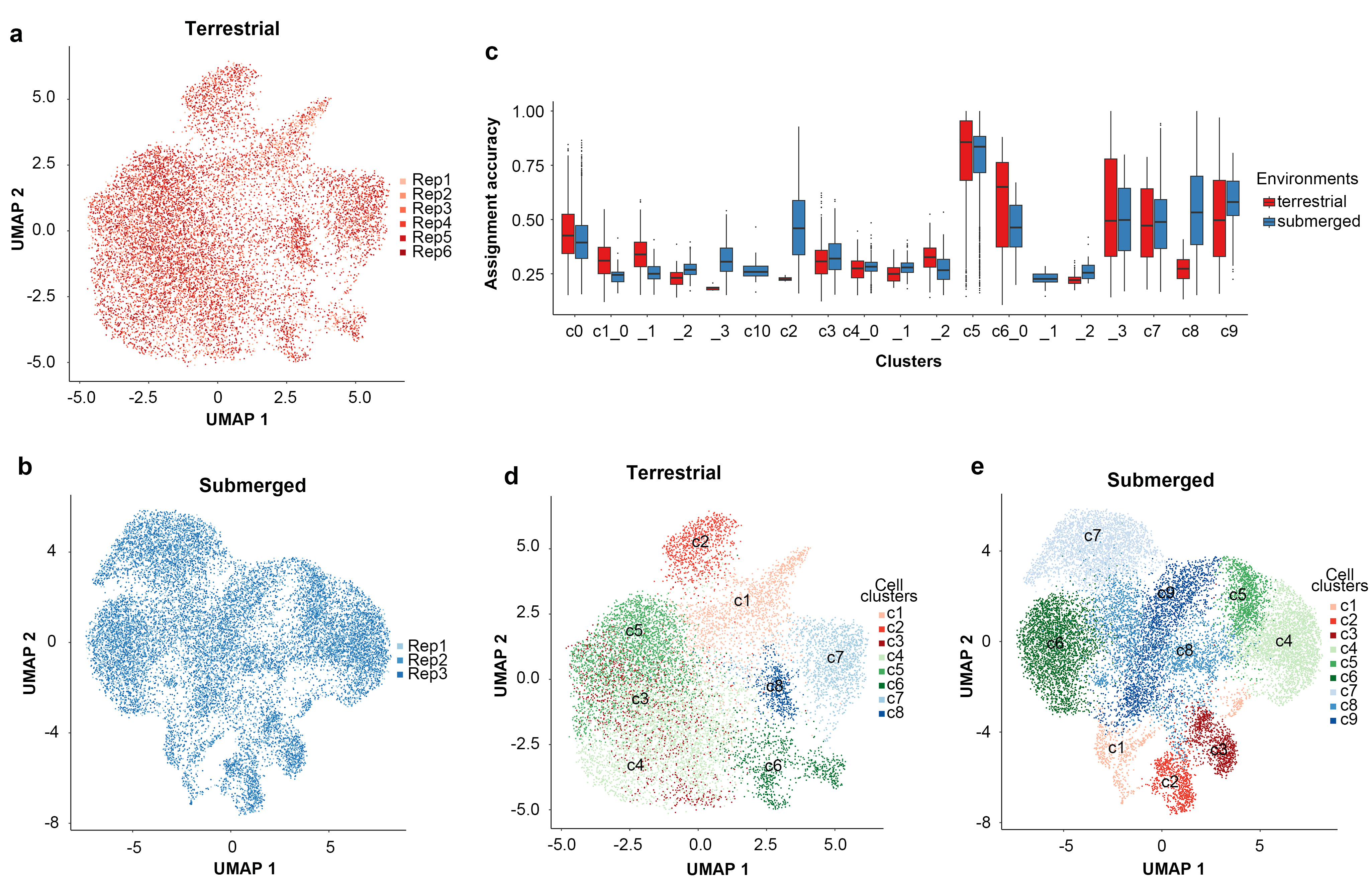


**Figure S12. Single-nucleus chromatin accessibility landscape in *E. baldwinii.***

(a,b) UMAP dimensional reduction projection for terrestrial (a) and submerged (b) culms. (c) The matched scores of snATAC-seq clusters and snRNA-seq profiles with ArchR. (d,e) Clusters for terrestrial (d) and submerged (e) culms, defined by a *de novo* method.

**
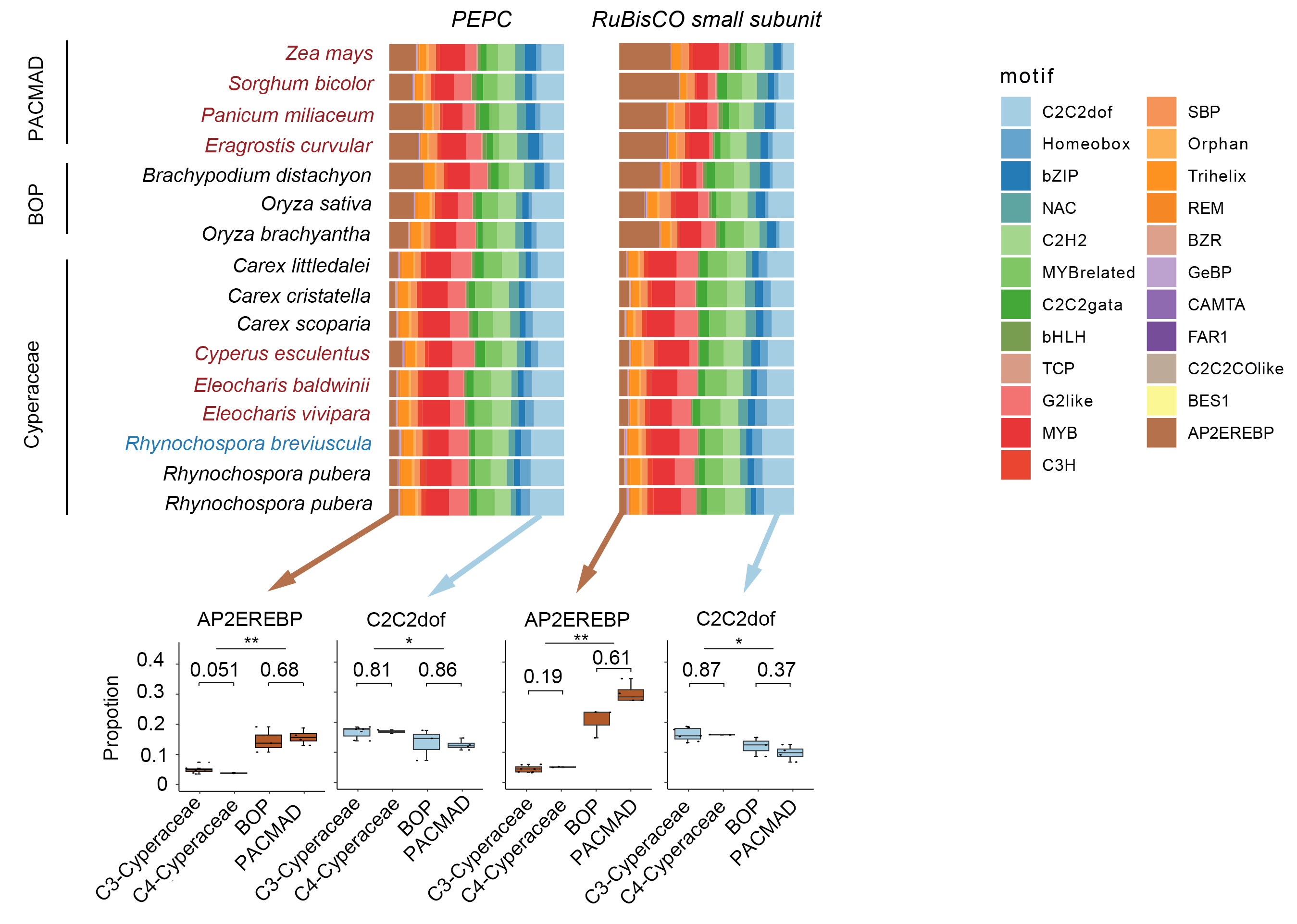
**

**Figure S13. The normalized abundance of motifs in promoter DNA sequences.** Cyperaceae, BOP, and PACMAD clades of Poaceae were included for the analyses. BOP: Bambusoideae, Oryzoideae, and Pooideae. PACMAD: Panicoideae, Aristidoideae, Chloridoideae, Micraioideae, Arundinoideae, and Danthonioideae. The C_4_ plants were highlighted in red; the plants only forming Kranz anatomy was in blue. In boxplots, the horizontal lines represent the median, and the vertical lines mark the range from the fifth and 95th percentile of the total data. The one-side t-test were performed. **P* values <0.05. ***P* values <0.01.

**
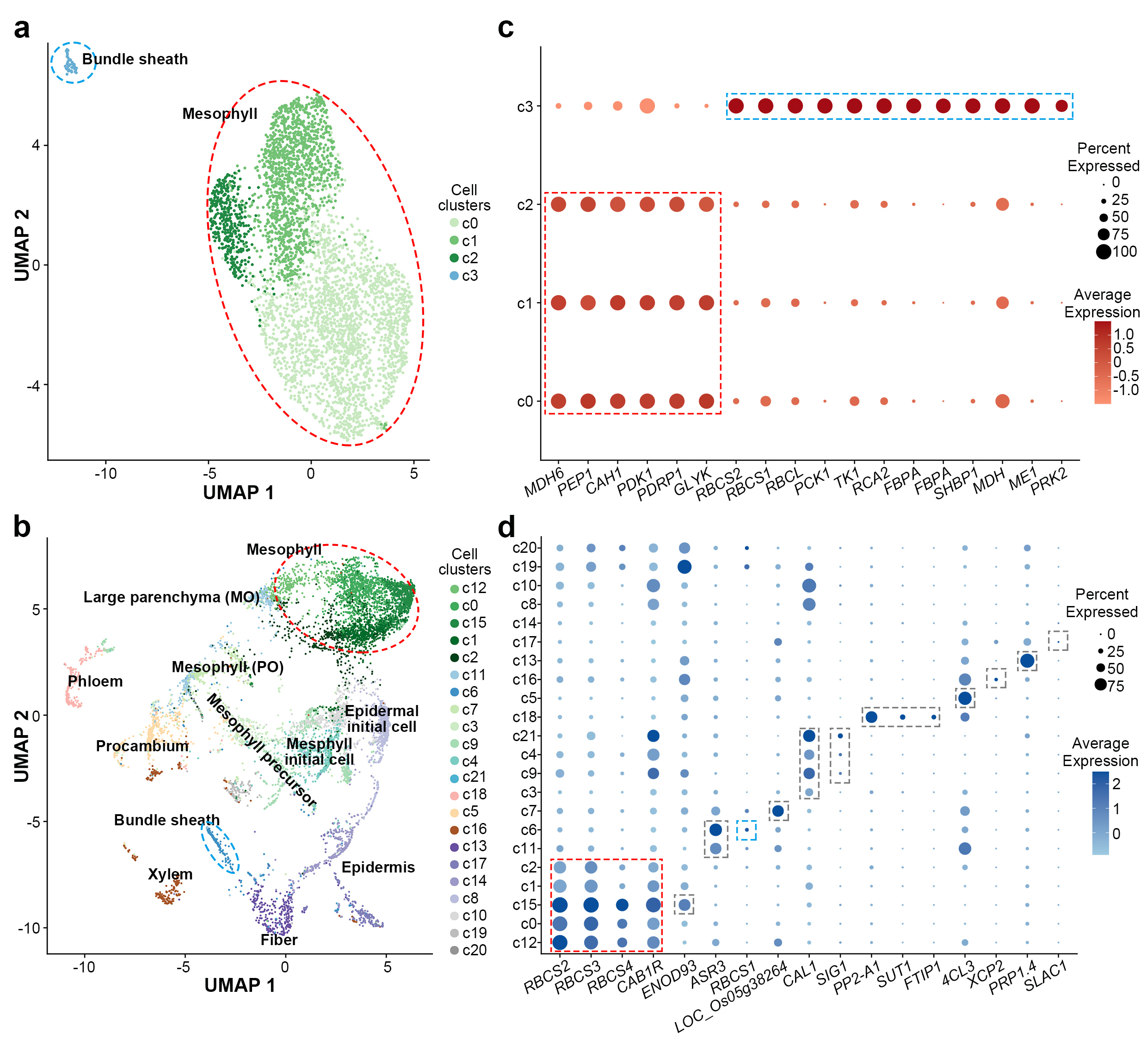
**

**Figure S14. Single-cell transcriptome atlas of maize and rice.**

(a,b) UMAP dimensional reduction projection of maize (a) and rice (b) leaf cells. (c-d) Dot plot showing the expression of representative markers that defined the major cell types for maize (c) and rice (d). Dashed box in the dot plot colored with "red" and "blue" represent the marker gene expression clusters belongs to MCs and BSCs, respectively. "grey" dashed box represented the markers in other cell types.


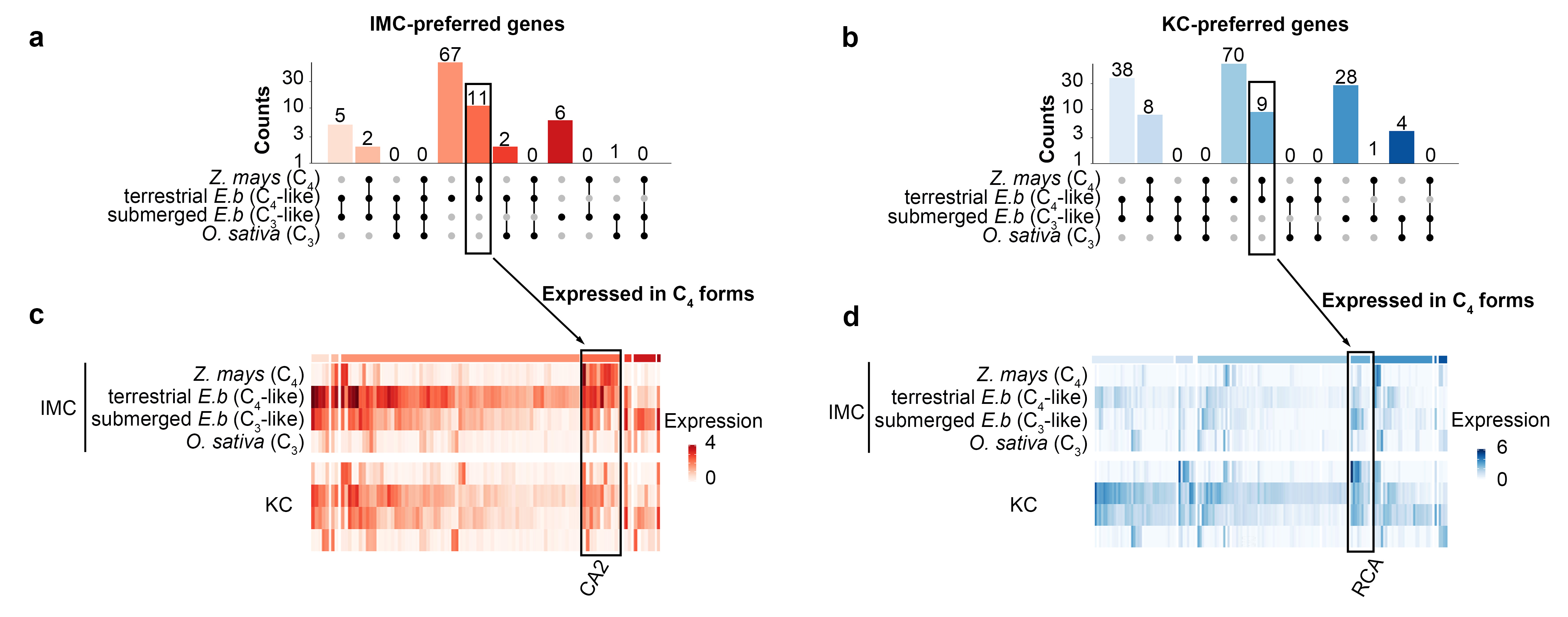


**Figure S15. Limited co-selections of** **cell-specialized genes across monocots.**

(a) The Upset plots illustrated the intersection of IMC-specialized genes across monocots. (b)The Upset plots illustrated the intersection of KC-specialized genes across monocots. (c) The heat maps showed the expression levels of these IMC- specialized genes. (d) The heat maps showed the expression levels of these KC- specialized genes. In (a) to (d), the black boxes highlighted the cell-specialized genes only in C_4_ and C_4_-like forms but not in C_3_ and C_3_-like forms. Orthologous gene of maize, rice, *E. baldwinii* were identified with reciprocal blast analysis (maize vs *E. baldwinii*, rice vs *E. baldwinii*), with e-value of 1e-10.

**Table S1. Summary of genome survey with GenomeScope2.**

| Property | Min | Max |
| --- | --- | --- |
| Homozygous (aaaa) | 90.54% | 93.45% |
| Heterozygous (not aaaa) | 6.55% | 9.46% |
| aaab | 0% | 0.87% |
| aabb | 6.11% | 7.17% |
| aabc | 0% | 0.52% |
| abcd | 0.45% | 0.90% |
| Genome Haploid Length | 489,150,822 bp | 490,486,157 bp |
| Genome Repeat Length | 245,392,251 bp | 246,062,148 bp |
| Genome Unique Length | 243,758,571 bp | 244,424,009 bp |
| Model Fit | 67.10% | 92.21% |
| Read Error Rate | 0.37% | 0.37% |

**Table S2. Basic statics of genome assembly and annotation to *E. baldwinii.***

| Estimated genome size (Gb) | 0.99 |
| --- | --- |
| Assembled genome size (Gb) | 1.06 |
| Number of contigs | 996 |
| N50 contig length (Mb) | 96.74 |
| GC content (%) | 35.85 |
| Number of genes | 43,113 |
| Number of transcripts | 65,431 |
| Size of retrotransposons (Mb) | 282.37 |
| Size of DNA transposons (Mb) | 253.43 |
| Other repeats (Mb) | 101.99 |
| Size of total repeat sequence (Mb) | 637.79 |
| BUSCO completeness of assembly (%) | 95.3 |
| LTR assembly index score | 18.56 |

**Table S3. Mapping statistics of NGS short reads to *E. baldwinii* genome.**

| Type | Statistics |
| --- | --- |
| Number of reads | 1,932,640,349 |
| Number of mapped reads | 1,925,930,845 |
| Number of pair-end reads in sequencing | 1,859,647,574 |
| Number of properly mapped pair-end reads | 1,699,382,154 |
| Coverage | 99.92% |
| coverage_high (avgerage depth >10) | 99.91% |
| Depth | 155.64 |
| Homozygous SNP | 84 |
| Heterozygous SNP | 426,688 |
| Homozygous INDEL | 343 |
| Heterozygous INDEL | 82,172 |

**Table S4. Statistics of transposable elements in *E. baldwinii* genome.**

| Repeat Classes |  |  |  |
| --- | --- | --- | --- |
| Total Sequences | 996 |  |  |
| Total Length | 1,062,083,314 bp |  |  |
| Class | Count | bpMasked | %masked |
| LTR |  |  |  |
| Copia | 86,267 | 83,150,984 | 7.83% |
| Gypsy | 90,158 | 144,171,476 | 13.57% |
| unknown | 86,166 | 53,924,618 | 5.08% |
| TIR |  |  |  |
| CACTA | 45,423 | 17,166,917 | 1.62% |
| Mutator | 126,034 | 39,648,038 | 3.73% |
| PIF_Harbinger | 11,547 | 2,727,207 | 0.26% |
| Tc1_Mariner | 3,299 | 1,635,173 | 0.15% |
| hAT | 75,154 | 28,910,515 | 2.72% |
| polinton | 235 | 346,780 | 0.03% |
| nonLTR |  |  |  |
| LINE_element | 1,477 | 899,846 | 0.08% |
| unknown | 381 | 220,654 | 0.02% |
| nonTIR |  |  |  |
| helitron | 458,500 | 162,999,986 | 15.35% |
| repeat_region | 72,440 | 101,988,682 | 9.60% |
| Total | 1,057,081 | 637,790,876 | 60.05% |
